# Combining gene expression analysis of gastric cancer cell lines and tumor specimens to identify biomarkers for anti-HER therapies—the role of HAS2, SHB and HBEGF

**DOI:** 10.1186/s12885-022-09335-4

**Published:** 2022-03-09

**Authors:** Karolin Ebert, Ivonne Haffner, Gwen Zwingenberger, Simone Keller, Elba Raimúndez, Robert Geffers, Ralph Wirtz, Elena Barbaria, Vanessa Hollerieth, Rouven Arnold, Axel Walch, Jan Hasenauer, Dieter Maier, Florian Lordick, Birgit Luber

**Affiliations:** 1 Technische Universität München, Fakultät für Medizin, Klinikum rechts der Isar, Institut für Allgemeine Pathologie und Pathologische Anatomie, 81675 München, Germany; 2 University Cancer Center Leipzig (UCCL), University Leipzig Medical Center, 04103 Leipzig, Germany; 3 Faculty of Mathematics and Natural Sciences, University of Bonn, 53113 Bonn, Germany; 4 Center for Mathematics, Technische Universität München, 85748 Garching, Germany; 5 Helmholtz Zentrum für Infektionsforschung, 38124 Braunschweig, Germany; 6 STRATIFYER Molecular Pathology GmbH, 50935 Köln, Germany; 7 Helmholtz Zentrum München-German Research Center for Environmental Health, Research Unit Analytical Pathology, 85764 Neuherberg, Germany; 8 Helmholtz Zentrum München-German Research Center for Environmental Health, Institute of Computational Biology, 85764 Neuherberg, Germany; 9 Biomax Informatics AG, 82152 Planegg, Germany

**Keywords:** Gastric cancer, Gene expression, Biomarker, HAS2, SHB, HBEGF

## Abstract

**Background:**

The standard treatment for patients with advanced HER2-positive gastric cancer is a combination of the antibody trastuzumab and platin-fluoropyrimidine chemotherapy. As some patients do not respond to trastuzumab therapy or develop resistance during treatment, the search for alternative treatment options and biomarkers to predict therapy response is the focus of research. We compared the efficacy of trastuzumab and other HER-targeting drugs such as cetuximab and afatinib. We also hypothesized that treatment-dependent regulation of a gene indicates its importance in response and that it can therefore be used as a biomarker for patient stratification.

**Methods:**

A selection of gastric cancer cell lines (Hs746T, MKN1, MKN7 and NCI-N87) was treated with EGF, cetuximab, trastuzumab or afatinib for a period of 4 or 24 h. The effects of treatment on gene expression were measured by RNA sequencing and the resulting biomarker candidates were tested in an available cohort of gastric cancer patients from the VARIANZ trial or functionally analyzed in vitro.

**Results:**

After treatment of the cell lines with afatinib, the highest number of regulated genes was observed, followed by cetuximab and trastuzumab. Although trastuzumab showed only relatively small effects on gene expression, *BMF*, *HAS2* and *SHB* could be identified as candidate biomarkers for response to trastuzumab. Subsequent studies confirmed *HAS2* and *SHB* as potential predictive markers for response to trastuzumab therapy in clinical samples from the VARIANZ trial. *AREG*, *EREG* and *HBEGF* were identified as candidate biomarkers for treatment with afatinib and cetuximab. Functional analysis confirmed that *HBEGF* is a resistance factor for cetuximab.

**Conclusion:**

By confirming *HAS2*, *SHB* and *HBEGF* as biomarkers for anti-HER therapies, we provide evidence that the regulation of gene expression after treatment can be used for biomarker discovery.

Trial registration.

Clinical specimens of the VARIANZ study (NCT02305043) were used to test biomarker candidates.

**Supplementary Information:**

The online version contains supplementary material available at 10.1186/s12885-022-09335-4.

## Background

Gastric cancer is the fifth most frequently diagnosed cancer and the fourth leading cause of cancer death worldwide [1]. In patients with locally advanced or metastatic disease, chemotherapy can prolong survival and reduce symptoms. The HER2-targeting antibody trastuzumab in combination with platin-fluoropyrimidine is the standard of care for patients with HER2 positive advanced gastric cancer [2]. Trastuzumab was approved following the Trastuzumab for Gastric Cancer (ToGA) trial showing a median overall survival of 13.8 month in patients receiving chemotherapy plus trastuzumab, compared to 11.1 month in patients receiving chemotherapy alone [3]. In contrast, the EGFR-targeting antibody cetuximab failed to improve survival in the randomised international Erbitux (cetuximab) in combination with Xeloda (capecitabine) and cisplatin in advanced esophago-gastric cancer (EXPAND) study [4]. However, subgroups of gastric cancer patients may benefit from anti-EGFR treatment. Therefore, biomarkers could help to identify those patients. The pan-HER tyrosine kinase inhibitor afatinib in combination with chemotherapy as first or second line therapy is currently being investigated in clinical trials [5–7]. First results from a small patient cohort are already available. 32 trastuzumab-resistant patients with HER2 positive metastatic esophageal, gastroesophageal junction or gastric adenocarcinoma were treated with either afatinib alone or the combination of trastuzumab and afatinib. The three patients with best changes in tumor volume demonstrated *EGFR* and *HER2* co-amplification in pretreatment tumor biopsies. Analysis of post-mortem metastatic samples in three patients who initially showed response to afatinib treatment, revealed loss of *EGFR* amplification and acquisition of *MET* amplification as mechanisms for acquired resistance [8]. The co-occurrence of alterations in *EGFR*, *MET*, *HER3*, *CCNE1*, *CDK6*, *CCND1* and *PIK3CA* in HER2-positive gastric carcinoma has been shown to confer resistance to HER2-targeted therapies in vitro [9]*.* Moreover, loss of *PTEN* and low *HER2* amplification correlated with trastuzumab resistance in 129 HER2-positive gastric cancer patients [10, 11]. These studies underline that not all patients respond to targeted therapies, and therapy resistance caused by bypass track mechanisms is one of the most common problems [2].

Biomarkers for anti-HER therapies are urgently required to select the appropriate treatment for gastric cancer patients. We hypothesize that the regulation of a gene by a specific treatment indicates its importance for treatment response and thus it might be used as biomarker for patient stratification. To this end we used gene expression analysis of gastric cancer cell lines to identify candidate biomarkers and validated our findings in cell culture or available clinical specimens [12–15].

## Methods

### Cell culture

The gastric cancer cell lines were provided by the following cell banks: MKN1 (Cell Bank RIKEN BioResource Center, Tsukuba, Japan, catalogue number RCB1003), MKN7 (Cell Bank RIKEN BioResource Center via tebu-bio, Offenbach, Germany, catalogue number JCRB1025), NCI-N87 (ATCC Cell Biology Collection via LGC Standards GmbH, Wesel, Germany, catalogue number, CRL-5822) and Hs746T (ATCC Cell Biology Collection via LGC Standards GmbH, Wesel, Germany, catalogue number ATCC HTB-135). The cell lines were cultured as described earlier [16–18].

Cell lines were selected according to the previously published response characterization already explained in Ebert et al*.* [18]. MKN1 cells are responsive to cetuximab treatment whereas Hs746T cells are not [16, 19]. NCI-N87 cells were described as trastuzumab responder and MKN7 and MKN1 cells as nonresponder. NCI-N87, MKN1 and MKN7 cells were described as afatinib responder while Hs746T cells were described as afatinib non-responder [17]. We have shown the HER2 positivity of NCI-N87 and MKN7 cells by immunohistochemistry before in Keller et al*.* (2018) [17], Fig. S1.

### RNA extraction

Cells were seeded in 10 cm dishes one day before treatment (cell numbers see Table S1, Additional file 1) and subsequently treated with EGF (5 ng/ml, Sigma Aldrich), cetuximab (Cet, 1 µg/ml, Merck), trastuzumab (Tra, 5 µg/ml, Roche), afatinib (Afa, 0.5 µM, Biozol) or dimethylsulfoxid (DMSO, 0.05%, afatinib solvent control) for 4 h or 24 h. RNA and micro RNA were isolated using the mirVana™ miRNA Isolation Kit (Thermo Fisher Scientific) and RNA was eluted in nuclease-free water. The DNA-free™ DNA Removal Kit (Thermo Fisher Scientific) was used for DNase digestion according to manufacturer’s instructions. All experiments were performed in triplicate.

The treatment times of 4 h and 24 h were chosen because of literature, previous experiments and duration of phenotypic analyses. The 4 h treatment was chosen because it corresponds to the middle of the film length of 7 h. The 24 h treatment was chosen since apoptosis was analyzed 24 h after treatment and effects on gene expression were shown in breast cancer cell lines after 24 h trastuzumab treatment [20]. Moreover, this time was chosen since previous gene expression experiments with cetuximab were performed after 24 h treatment.

### Next generation sequencing and primary data analysis

The dataset of differently expressed genes resulting from next generation sequencing was published previously. Thus, regarding next generation sequencing and primary data analysis we refer to Ebert et al*.* [18].

### Quantitative PCR

RNA was transcribed into cDNA using the High-Capacity cDNA Reverse Transcription Kit (Thermo Fisher Scientific). Candidate gene expression was measured using the TaqMan Gene Expression Assays for Amphiregulin *AREG* (Hs00950669_m1), Epiregulin *EREG* (Hs00914313_m1), Heparin Binding EGF Like Growth Factor *HBEGF* (Hs00181813_m1), Bcl-2 modifying factor *BMF* (Hs00372937_m1), Hyaluronan Synthase 2 *HAS2* (Hs00193435_m1), Src Homology-2 domain *SHB* (Hs00182370_m1), β-Actin *ACTB* (Hs01060665_g1, reference) and the TaqMan Universal PCR Master Mix (Thermo Fisher Scientific). All procedures were carried out according to manufacturer’s instructions. The LightCycler® 480 instrument and software (Roche) were used to determine the relative gene expression.

### ELISA

Cells were prepared in the same way as for RNA extraction. Conditioned medium was collected 24 h after treatment. HBEGF, AREG and EREG secretion was measured by ELISA (Human HB-EGF DuoSet ELISA, R&D Systems; Human Amphiregulin DuoSet ELISA, R&D Systems; Human Epiregulin ELISA Kit, Abcam) according to manufacturer’s instructions.

### Transfection with siRNA

Medium was exchanged to antibiotic-free medium one day after plating (cell numbers see Table S1, Additional file 1). Cells were transfected using Lipofectamine 2000 (Thermo Fisher Scientific) and HBEGF siRNA (as described [18]) or AREG siRNA (Flexi Tube Gene Solution (pool of 4 different siRNAs), Qiagen) two hours after medium replacement. As reported previously, the unlabeled and labeled (AF 488) All Star Negative Control siRNA (Qiagen) were used as controls [18]. Cells were plated for proliferation assay 24 h after transfection. RNA was extracted on day 1 and day 5 after transfection (RNeasy Mini Kit, Qiagen) to check the knockdown efficiency by qPCR. The efficiency was assessed with AF 488-labeled negative control siRNA one day after transfection. As described before, more than 90% of both MKN1 and NCI-N87 cells were successfully transfected [18].

### WST-1 proliferation assay

The water-soluble tetrazolium (WST-1) proliferation assay (Roche Diagnostics) was used to measure cell proliferation after knockdown or stimulation as described earlier [17]. Cells were treated with cetuximab (1/10 µg/ml, Merck), trastuzumab (5/20 µg/ml, Roche), afatinib (0.5 µM, Biozol), DMSO (0.05%, afatinib solvent), trastuzumab solvent (described in [17]) or cetuximab solvent (8.48 mg/ml NaCl, 1.88 mg/ml Na_2_HPO_4_ × 7H_2_O, 0.41 mg/ml NaH_2_PO_4_xH_2_O) for 72 h (cell numbers see Table S1, Additional file 1). In case of stimulation, cells were treated with 5 ng/ml recombinant HBEGF or 15 ng/ml recombinant AREG (R&D Systems).

### Statistical analyses for in vitro experiments

Each experiment was repeated at least three times. Data are presented as mean with standard deviation. SPSS Statistics (IBM) was used to calculate one-sample or two-sample t-test. The significant differences are indicated by *p < 0.05, **p < 0.01 or ***p < 0.001. For RNA sequencing data, the fold-change was log2-transformed (log2FC) and the p-value was adjusted according to Benjamini-Hochberg (FDR, p.adjust).

### Clinical study design

In the prospective, observational study VARIANZ (NCT02305043) 548 patients were recruited in 35 sites [12–15]. Patients received medical treatment for histological confirmed stage IV metastatic gastric or gastroesophageal junction adenocarcinoma (mGC/mGEJC). HER2 status was determined in central pathology by immunohistochemistry (IHC) and chromogenic in situ hybridization (CISH) as defined by ToGA study [3]. Patients were followed up to 48 months and trastuzumab treatment was recorded. The treatment decision was based on HER2 status assessed by local pathologies (59 patients HER2 positive, 40 patients HER negative, 1 patient unknown). For 100 patients RNA was extracted from formalin-fixed paraffin-embedded (FFPE) tissue as described in [21]. The 100 FFPE tissue samples consisted of 49 pre-therapeutic biopsies (29 from patients receiving trastuzumab, 20 from patients not receiving trastuzumab), 39 resection specimens (20 from patients receiving trastuzumab, 19 from patients not receiving trastuzumab) and 12 metastases (6 from patients receiving trastuzumab, 6 from patients not receiving trastuzumab). RT-qPCR was applied for relative quantification of BMF, HAS2 and SHB mRNA as well as CALM2 (calmodulin 2; housekeeping gene) expression by using gene-specific TaqMan®-based assays [22]. Forty amplification cycles were applied and the cycle quantification threshold (CT) values of marker genes and the reference gene for each sample were estimated as the median of the triplicate measurements. The final values were generated by using ΔCT from the total number of cycles. The relative expression levels of the target transcripts were calculated as 40 – DCT values (DCT = mean CT target gene – mean CT housekeeping gene) to yield positively correlated numbers and to facilitate comparisons. This ensures that high normalized gene expression values obtained by the test are proportional to the high gene expression levels.

### Statistical analyses of clinical data

The survival analysis was carried out using the Kaplan–Meier estimation and Cox regression analysis available in Matlab R2016b (ecdf and coxphfit, respectively). For the Cox regression analysis, the 95% confidence interval was calculated for the estimated hazard ratios (HR) to determine significance. HR > 1 indicates high expression group patients have low survival, HR < 1 suggests high survival and HR = 1 indicates a lack of association with survival. The variable adjusted in the Cox regression was the classification as high or low expression, given an optimal gene expression cut-off value. The optimal gene expression cut-off value was used to divide the patients into high- and low-risk groups. This was obtained by fitting the Cox regression model with a range of plausible gene expression cut-off values and by selecting the one providing lowest Cox regression p value as the optimal one. This was performed individually for each considered gene (*HAS2, SHB* and *BMF*). We defined gene expression values higher or equal to the optimal cut-off value as high expression, while lower values were defined as low expression. For the Kaplan–Meier estimation, significant differences between patient groups were assessed using the log-rank test.

## Results

### Differential gene expression

**Fig. 1:**
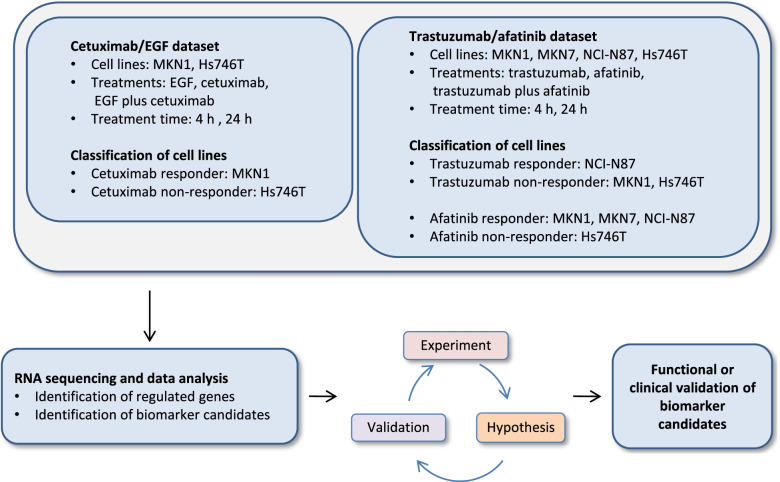
Workflow for gene expression analysis with identification and validation of candidate biomarkers. Gastric cancer cell lines were treated with EGF, cetuximab, EGF plus cetuximab, trastuzumab, afatinib or trastuzumab plus afatinib. The classification of cell lines into responders and non-responders was carried out previously: MKN1 cells were responsive to cetuximab treatment, Hs746T cells were non-responsive [16, 19]. NCI-N87 cells were trastuzumab-responsive, MKN7 and MKN1 cells were non-responsive. NCI-N87, MKN1 and MKN7 cells were afatinib-responsive, Hs746T cells were non-responsive [17]. Regulated genes and biomarker candidates were identified following gene expression analysis. Biomarker candidates were validated in cell culture or clinical specimens

The workflow for gene expression and functional analysis is illustrated in Fig. 1. Genes with log2-fold-change (log2FC) > 1 or < -1 and false discovery rate (FDR) < 0.05 were selected to identify those that were regulated after each treatment or are differentially expressed in different cell lines (Tables S2-S5, Additional file 1). The functional enrichment analysis for this dataset was already described in Ebert et al*.* [18].

The hypothesis that the regulation of a gene by a specific treatment indicates its importance for treatment response was validated in cell culture or available clinical specimens.

#### Cetuximab treatment changes gene expression in MKN1 cells

We analyzed the gene expression profiles of MKN1 (cetuximab responder) and Hs746T cells (cetuximab non-responder) [16, 19] after 4 h or 24 h cetuximab and/or EGF treatment. We used EGF and cetuximab treatment as we wanted to compare the transcriptional changes of a treatment inducing phenotypic response, namely EGF, with a treatment inhibiting this response i.e. cetuximab. Differential gene expression results for MKN1 cells are listed in Table S2 (Additional file 1). The number of differentially expressed genes generally increased between the 4 h and 24 h time points (compare *rows 12/17 (Cetuximab) and rows 14/19 (EGF))*. EGF showed a stronger effect on gene expression than cetuximab (compare rows 12/14 (24 h) and rows 17/19 (4 h). Cetuximab and EGF did not influence the gene expression profile of Hs746T cells (not shown).

#### Trastuzumab treatment changes gene expression in NCI-N87 cells

Following treatment with trastuzumab for 4 h and 24 h no genes were regulated in the responder cell line NCI-N87 [17], according to the selection criteria (log2FC > 1 or < -1 and FDR < 0.05). Nevertheless, we identified 3 genes (*SHB, HAS2, BMF*) that had either a logFC or FDR close to the selection criteria (**Table ****1**). Trastuzumab did not affect gene expression in MKN7, MKN1 or Hs746T cells.

**Table 1:** Regulated genes after 4 h or 24 h trastuzumab treatment in NCI-N87 cells

	**NCI-N87_4h_Tra vs. NCI-N87_4h_untr**		**NCI-N87_24h_Tra vs. NCI-N87_24h_untr**	
**Gene Symbol**	**log2FC**	**FDR**	**log2FC**	**FDR**
*HAS2*	0.2	0.999	**-0.9**	**0.246**
*SHB*	**0.6**	**0.058**	-0.1	0.996
*BMF*	-0.2	0.999	**0.7**	**0.009**

Following trastuzumab treatment no genes were regulated according to the selection criteria. The conditions with log2FC or FDR close to the selection criteria are indicated in bold

#### Afatinib treatment changes gene expression in NCI-N87, MKN7 and MKN1 cells

The gene expression profile was analyzed in the afatinib responder cell lines NCI-N87, MKN1 and MKN7 and the afatinib non-responder cell line Hs746T [17]. Differential gene expression results following afatinib treatment are listed in Tables S2-S4 (Additional file 1). The number of differentially expressed genes generally increased between the 4 h and 24 h time points (compare rows 2/7 of Table S2 (MKN1), rows 2/7 of Table S3, (NCI-N87) and rows 2/7 of Table S4 (MKN7)). Afatinib had the strongest effect on gene expression in NCI-N87 cells, followed by MKN7 and MKN1 cells (compare row 2 of Tables S2, S3 and S4 (24 h) and row 7 of Tables S2, S3 and S4 (4 h)). Afatinib did not affect gene expression in Hs746T cells (not shown).

#### Gene expression changes are similar after trastuzumab plus afatinib and afatinib treatment

The gene expression profile of NCI-N87, MKN1, MKN7 and Hs746T cells following trastuzumab plus afatinib treatment was analyzed. The numbers of differentially expressed genes are listed in Tables S2-S4 (Additional file 1). Since trastuzumab alone had only a marginal effect on gene expression in NCI-N87 cells, its impact in combination with afatinib was investigated. The scatter plot was generated to compare the genes that were regulated after the combination treatment only. The genes that were regulated after trastuzumab plus afatinib treatment but were not regulated after afatinib treatment are highlighted as red dots in the scatter plot. The red dots are all close to a logFC of 1 and -1, respectively. Thus, there is no clear difference between genes that were regulated by trastuzumab plus afatinib and genes that were regulated by afatinib only (Fig. S1, Additional file 2).

### Identification of biomarker candidates

#### Candidate biomarkers for cetuximab treatment were identified

**Fig. 2:**
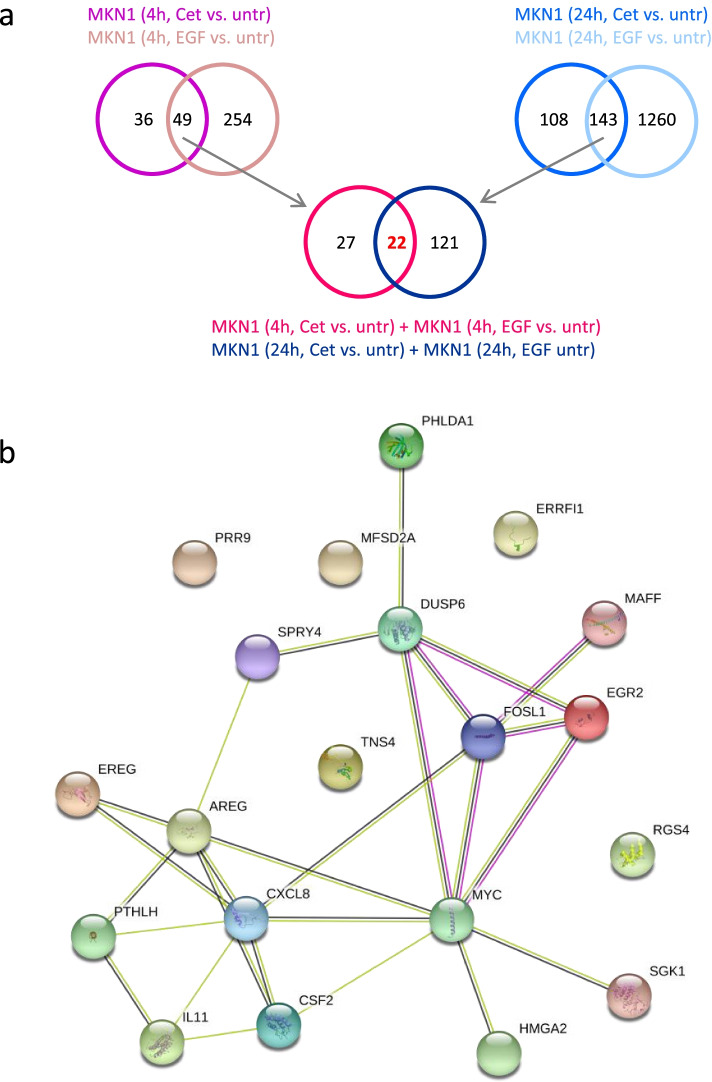
Identification of candidate biomarkers for cetuximab treatment in MKN1 cells. **a** 49 genes were regulated after 4 h cetuximab and EGF treatment whereas 143 genes were regulated after 24 h cetuximab and EGF treatment. The 22 genes which were regulated after 4 h and 24 h cetuximab and EGF treatment were identified as candidate biomarkers. **b** The 22 genes which were regulated by cetuximab as well as by EGF treatment after 4 h and 24 h were analyzed using the STRING tool. The colors indicate different functional associations (green: textmining, black: co-expression, pink: experimentally determined)

We hypothesized that genes that are inversely regulated by the EGFR ligand EGF and the EGFR antibody cetuximab might be candidate biomarkers for cetuximab response. In total, 22 genes were regulated after 4 h and 24 h EGF and cetuximab treatment. Of note, only the genes that were regulated by EGF as well as by cetuximab after 4 h and 24 h are depicted (Fig. 2 a, Table S6, Additional file 2). Amongst them are genes that regulate MAPK signaling (*DUSP6, SPRY4*), EGFR ligands Amphiregulin and Epiregulin (*AREG, EREG*), transcription factors (*FOSL1, MYC, EGR2*), cytokines (*CSF2, IL11, IL8*) and the HER-family feedback inhibitor *ERRFI1*. All of the genes listed in Table S6 (Additional file 1) were upregulated by EGF and downregulated by cetuximab. One exception is *EGR2,* which was downregulated after 24 h EGF treatment but upregulated after 4 h EGF treatment. The EGFR ligand Heparin Binding EGF Like Growth Factor *HBEGF* was significantly regulated following 4 h and 24 h EGF and 24 h cetuximab treatment but not following 4 h cetuximab treatment. Thus, HBEGF was filtered out according to the selection criteria. Since the threshold for significance was nearly achieved (FDR 0.075) and we were especially interested in regulation of EGFR ligands, we considered *HBEGF* as additional candidate biomarker. The analysis of functional protein association networks provided by the STRING tool revealed connections between the candidate biomarkers *EREG, AREG, PTHLH, IL8 (CXCL8), IL11, CSF2, MYC, HMGA2, SGK1, FOSL1, MAFF, EGR2, DUSP6, PHLDA1* and *SPRY4* ((https://string-db.org*)* [23]). Moreover, the analysis revealed *MYC, IL8, FOSL1* and *DUSP6* as central hubs, showing many connections to other candidate biomarkers (Fig. 2b).

#### Candidate biomarkers for trastuzumab treatment were identified

Since only 3 genes encoding Src Homology-2 domain, Hyaluronan Synthase 2 and Bcl-2 modifying factor (*SHB, HAS2, BMF*) that had either a logFC or FDR close to the selection criteria were observed, no additional narrowing of the biomarker candidates for trastuzumab response was necessary (Table 1).

#### Candidate biomarkers for afatinib treatment were identified

**Fig. 3:**
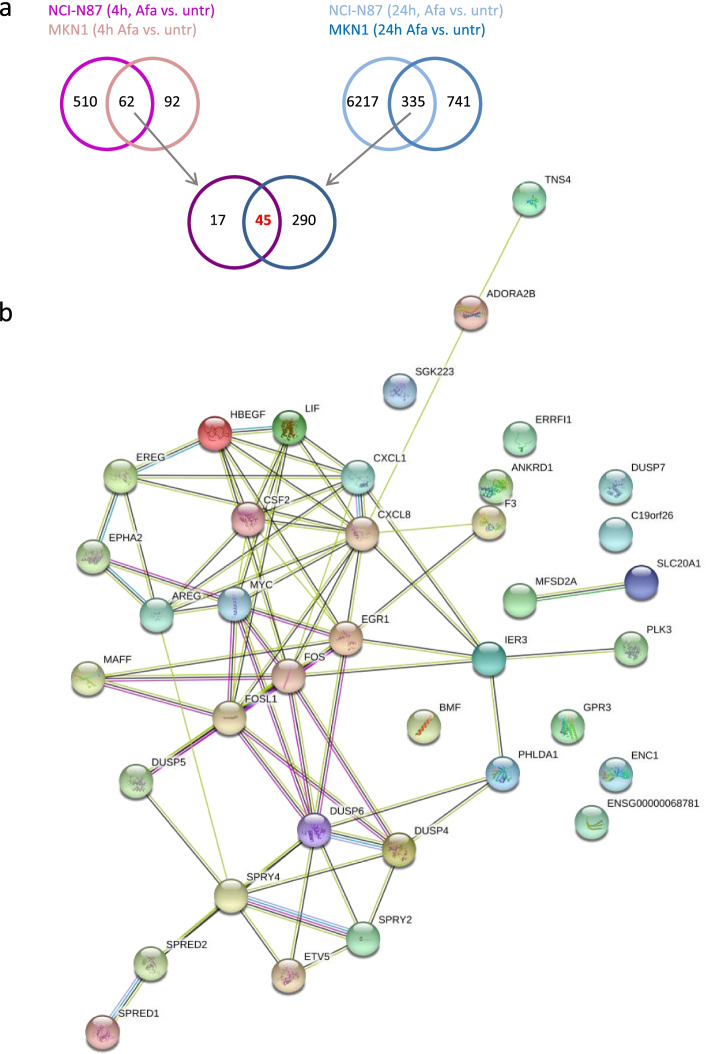
Identification of candidate biomarkers for afatinib treatment in NCI-N87 and MKN1 cells. **a** 62/335 genes were regulated after 4 h/24 h afatinib treatment in NCI-N87 and MKN1 cells. The 45 genes that were regulated after 4 h and 24 h afatinib treatment in NCI-N87 and MKN1 cells were identified as candidate biomarkers. **b** The 45 genes which were regulated in NCI-N87 as well as in MKN1 cells after 4 h and 24 h afatinib treatment were analyzed using the STRING Tool. The colors indicate different functional associations (green: textmining, black: co-expression, blue: from curated databases, pink: experimentally determined, purple: protein homology)

In order to isolate robust biomarkers for afatinib response we extracted genes that were regulated at two time points in two responder cell lines. The MKN7 cell line was excluded from this analysis because of its weak response in the proliferation assay [17]. The 45 genes that were regulated after 4 h and 24 h afatinib treatment in NCI-N87 and MKN1 cells were considered as candidate biomarkers. Of note, only genes that were regulated in NCI-N87 as well as in MKN1 cells after 4 h and 24 h treatment were depicted (Fig. 3a, Table S7, Additional file 1). Amongst these are genes that regulate MAPK signaling (*DUSP4, DUSP5, DUSP6, DUSP7, SPRY4*), EGFR ligands (*AREG, EREG, HBEGF*), transcription factors (*FOSL1, MYC, EGR2*), cytokines (*CSF2, IL11, IL8*), the apoptosis regulator *BMF* and the HER-family feedback inhibitor *ERRFI1*. Most of the 45 genes, except *BMF, STON1-GTF2A1L, AL590560.1, AC027117.2,* were downregulated after afatinib treatment. The analysis of functional protein association networks, using the STRING tool, revealed connections between the candidate biomarkers *SPRED1, SPRED2, SPRY4, SPRY2, DUSO4, DUSP5, DUSP6, PHLDA1, IER3, PLK3, FOS, FOSL1, MAFF, EGR1, F3, IL8 (CXCL8), IL1 (CXCL1), MYC, AREG, EPHA2, EREG, HBEGF, LIF, CSF2, ADORA2B* and *TNS4* ((https://string-db.org*)* [23]). *DUSP6, FOS, EGR1, MYC* and *IL8 (CXCL8)* were identified as central hubs showing many connections to other genes (Fig. 3b).

#### Comparison of candidate biomarkers for cetuximab and afatinib response

The candidate biomarkers *AREG, EREG, HBEGF* and the central hubs *DUSP6, MYC* and *IL8* were regulated in the cetuximab responder and both afatinib responder cell lines (Figs. 2 and 3).

### Regulated genes were confirmed by qPCR and ELISA

**Fig. 4:**
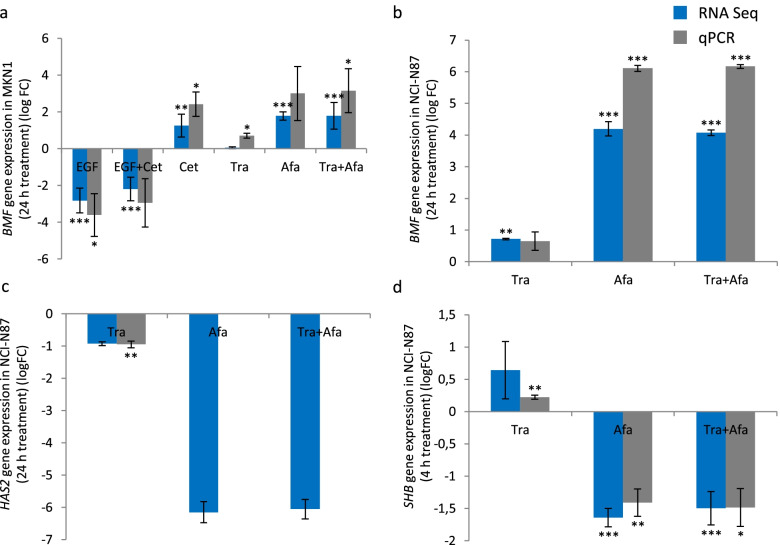
Confirmation of trastuzumab candidate biomarkers *BMF, HAS2*, and *SHB*. MKN1 **a** cells were treated with EGF, EGF plus cetuximab (EGF + Cet), cetuximab (Cet), trastuzumab (Tra), afatinib (Afa) or trastuzumab plus afatinib (Tra + Afa). NCI-N87 **b**, **c**, **d** were treated with trastuzumab (Tra), afatinib (Afa) or trastuzumab plus afatinib (Tra + Afa). The selected treatment times were 24 h for BMF **a**, **b** and HAS2 **c** and 4 h for SHB **d**. *BMF* **a**, **b**, *HAS2* **c**, *SHB* **d** gene expression was measured by RNA Sequencing and qPCR. The mean of three biological experiments with standard deviation is shown. Statistically significant effects compared to untreated are indicated by *p < 0.05, **p < 0.01 or ***p < 0.001 (one-sample t-test)

Seven selected genes were validated by qPCR for one treatment time. Three of them were also analyzed on protein level by ELISA. The gene expression levels of *AREG, EREG, HBEGF, BMF, SHB, HAS2* and CD274 (PD-L1) were qualitatively confirmed. The Pearson correlation coefficient ranged from 0.9877 to 1.000 whilst the Benjamini–Hochberg False Discovery Rate adjusted p-value ranged from 3.14E-06 to 0.0509 in NCI-N87, MKN1 and MKN7 cells (for FDR <  = 0.05). Due to the absence of any treatment effects, no correlations were observed in Hs746T cells (Table S8, Fig. 4, Fig. S1 and S2, Additional files 1 and 2, data for *HBEGF* and *CD274* (PD-L1) were published previously [18]).

The afatinib solvent DMSO was used in the validation experiments. No changes in gene expression were observed after DMSO treatment, except for minor changes on *SHB* expression in NCI-N87 cells. Of note, the mentioned effects of DMSO were in opposite direction than that of afatinib. Consequently, the effects we observed after afatinib treatment are caused by afatinib itself and not by its solvent DMSO (Fig. S4-S6, Additional file 2).

Additionally, we validated the RNA sequencing results on protein level. The conditioned medium after 24 h treatment was used in ELISA assays to detect the presence of secreted AREG, EREG and HBEGF. The levels of secreted EREG and HBEGF were below detection limit in untreated MKN1, NCI-N87, MKN7 and Hs746T cells. HBEGF was measurable in EGF-treated MKN1 cells only. In contrast, the AREG secretion was measurable in all conditions. The *AREG* gene expression levels measured by qPCR and RNA sequencing in MKN1, NCI-N87 and MKN7 cells were qualitatively confirmed with the exception of cetuximab-treated MKN1 cells (Table S8, Fig. S7, Additional files 1 and 2).

### Functional validation of biomarker candidates HBEGF and AREG

The HER-family ligands *HBEGF* and *AREG* were identified as candidate biomarkers for cetuximab and afatinib treatment. Since no suitable cohort was available for clinical validation, we performed in vitro knockdown and stimulation experiments to assess the importance of *HBEGF* and *AREG* for treatment sensitivity in MKN1 and NCI-N87 cells.

#### Importance of HBEGF for cetuximab but not afatinib response was confirmed by functional analysis

**Fig. 5:**
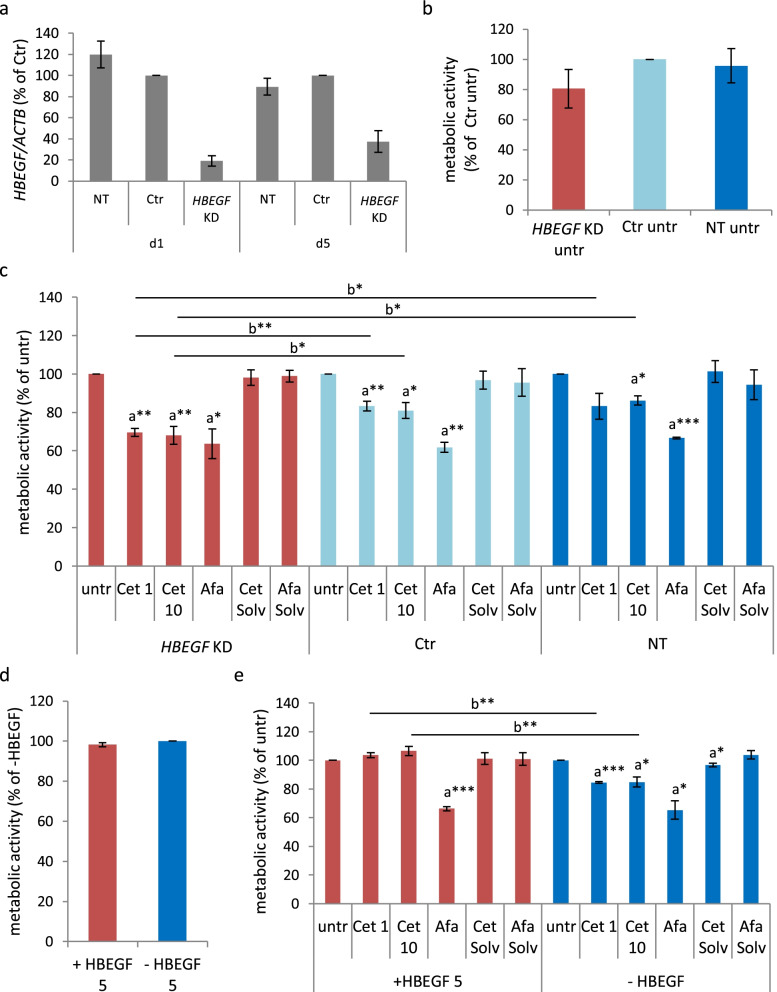
Effects of cetuximab and afatinib on proliferation after *HBEGF* knockdown or stimulation in MKN1 cells. MKN1 cells were transfected with negative-control (Ctr) or *HBEGF* (*HBEGF* KD) siRNA. Non-transfected (NT) cells were used as control. The knockdown was checked on RNA level on day 1 (d1) and day 5 (d5) after transfection **a**. The metabolic activity was measured by WST-1 proliferation assay for 72 h in the untreated **b** and treated **c** state. MKN1 cells were stimulated with 5 ng/ml HBEGF (+ HBEGF 5) or not stimulated (-HBEGF 5). The metabolic activity was measured by WST-1 proliferation assay for 72 h in the untreated **d** and treated **e** state. Cells were treated with 1 μg/ml cetuximab (Cet 1), 10 μg/ml cetuximab (Cet 10), 0.5 μM afatinib (Afa) or the corresponding solvents (Cet Solv, Afa Solv) for 72 h. Shown are the mean values ​​from three experiments with standard deviation. Significant effects compared to untreated within a group (*HBEGF* KD, Ctr, NT (**c**) or + HBEGF 5, -HBEGF 5 (**e**)) are indicated by *p < 0.05, a** p < 0.01 or a***p < 0.001 (one-sample t-test). Significant effects compared to Ctr, NT (c) or –HBEGF (e) with the same treatment are indicated by b* p < 0.05 or b** p < 0.01 (two-sample t-test)

*HBEGF* gene expression was reduced to 20% on day 1 and to 37% on day 5 after transfection in MKN1 cells (Fig. 5a). The proliferation assay was conducted during this time period. The metabolic activity was slightly reduced after *HBEGF* knockdown (*HBEGF* KD) compared to negative-control siRNA (Ctr) and non-transfected (NT) cells (Fig. 5b). In *HBEGF* KD cells, cetuximab reduced the metabolic activity to 70% (1 µg/ml Cet) and 68% (10 µg/ml Cet). Cetuximab reduced the metabolic activity to 83% (1 µg/ml Cet) and 81% (10 µg/ml Cet) in Ctr cells and to 83% (1 µg/ml Cet) and 86% (10 µg/ml Cet) in NT cells. The difference in cetuximab sensitivity between *HBEGF* KD and Ctr/NT cells was statistically significant. Afatinib reduced the metabolic activity to 64% (*HBEGF* KD), 62% (Ctr) and 67% (NT), respectively (Fig. 5c). The stimulation with 5 ng/ml HBEGF did not alter the metabolic activity of untreated MKN1 cells (Fig. 5d). While cetuximab reduced the metabolic activity to 85% (1/10 µg/ml Cet) without HBEGF stimulation this did not occur in presence of simultaneous HBEGF stimulation. The difference in cetuximab sensitivity observed between HBEGF-treated and non-treated MKN1 cells was statistically significant. Afatinib decreased the metabolic activity of the cells to 65%, irrespective of HBEGF treatment (Fig. 5e).

In NCI-N87 cells, *HBEGF* was reduced to 21% on day 1 after transfection and to 56% on day 5 after transfection (Fig. S8a, Additional file 2). The transfection itself reduced the metabolic activity in untreated NCI-N87 cells, regardless of the presence of *HBEGF* or negative-control siRNA (Fig. S8b, Additional file 2). Trastuzumab reduced the metabolic activity to 77% (5/20 µg/ml Tra) in *HBEGF* KD cells, to 90% (5 µg/ml Tra) or 83% (20 µg/ml Tra) in Ctr cells and to 87% (5 µg/ml Tra) or 78% (20 µg/ml Tra) in NT cells. Afatinib decreased the metabolic activity to 19% (*HBEGF* KD), 21% (Ctr) or 18% (NT), respectively. Thus, *HBEGF* knockdown did not affect trastuzumab or afatinib sensitivity in NCI-N87 cells (Fig. S8c, Additional file 2). The stimulation with HBEGF slightly increased the metabolic activity in untreated NCI-N87 cells (Fig. S9a, Additional file 2). The metabolic activity was reduced to 89% (5 µg/ml Tra) or 90% (20 µg/ml Tra) with simultaneous HBEGF stimulation and to 81% (5 µg/ml Tra) and 84% (20 µg/ml Tra) without HBEGF stimulation. Afatinib reduced the metabolic activity to 13% in presence of simultaneous HBEGF treatment, and to 19% without simultaneous HBEGF treatment. Although the difference in afatinib sensitivity after HBEGF stimulation is quite small, it was indeed significant (Fig. S9b, Additional file 2).

#### Importance of AREG for cetuximab and afatinib response was not confirmed by functional analysis

The same experiments were carried out for *AREG*. However, neither *AREG* knockdown nor AREG stimulation affected the proliferation or the sensitivity to cetuximab, trastuzumab, or afatinib (Fig. S10-S13, Additional file 2).

### *HAS2* and *SHB* are predictive risk factors for trastuzumab therapy response

**Fig. 6:**
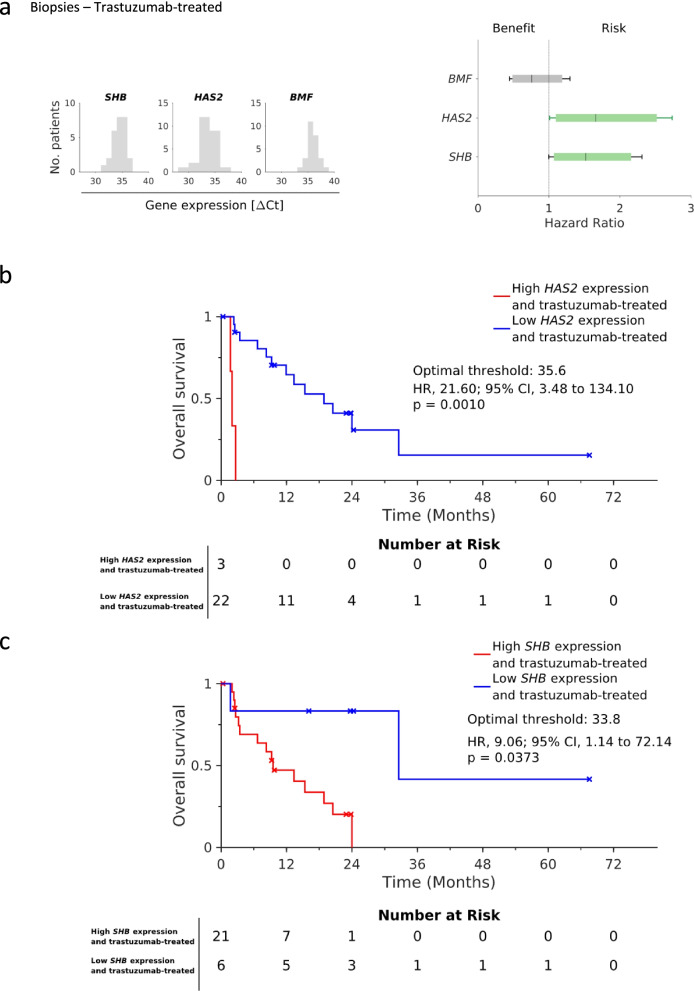
Identification of predictive biomarkers for overall survival in pre-therapeutic tumor biopsies from trastuzumab-treated patients. **a** Distribution of gene expression values and Hazard ratios (HR) obtained for *SHB* (HR 1.5; 95% CI, 1 to 2.31), *HAS2* (HR 1.7; 95% CI, 1.01 to 2.73) and *BMF* (HR 0.8; 95% CI, 0.44 to 1.3). *SHB* and *HAS2* are risk factors for patient overall survival. The error bars show the 95% and boxes the 90% confidence interval. Green coloring shows significance. **b** Kaplan–Meier curves show the overall survival of patients with respect to *HAS2* gene expression levels. Lower *HAS2* gene expression measured in tumor biopsies is beneficial, whereas higher *HAS2* gene expression is detrimental for patient overall survival under trastuzumab treatment. **c** Kaplan–Meier curves show the overall survival of patients in relation to *SHB* gene expression. Lower *SHB* gene expression measured in tumor biopsies is beneficial, whereas higher *SHB* gene expression is detrimental for patient overall survival under trastuzumab treatment. The method employed to obtain the p values was the log-rank test

The gene expression of trastuzumab candidate biomarkers *SHB, HAS2* and *BMF* was measured in 100 samples from the VARIANZ cohort (one sample from each patient), including pre-therapeutic biopsies, resection specimens and metastases [12–15] (see Methods section). The patient characteristics are shown in Table 2. Using a Cox-proportional hazards model, *HAS2* (hazard ratio (HR) 1.7, 95% confidence interval (CI) 1.01–2.73) and *SHB* (HR 1.5, 95% CI 1–2.31) were significant risk factors in biopsies of trastuzumab-treated patients. Moreover, *BMF* was not identified as beneficial or risk factor (HR 0.8, 95% CI 0.44–1.3) (Fig. 6a). The Kaplan–Meier curves showed an overall survival benefit for trastuzumab-treated patients with *HAS2* (log-rank test p = 0.0010) or *SHB* (log-rank test p = 0.0373) expression in biopsies below the optimized threshold (Fig. 6b and c). Kaplan–Meier curves using the quantile threshold 75% for *HAS2* and 25% for *SHB* as cut-offs are depicted in Fig. S14, (Additional file 2). These effects of *HAS2* and *SHB* were not observed in resection specimens or all tumor types of trastuzumab treated patients (Fig. S15a, b and c, Additional file 2). To clarify whether *HAS2, SHB* and *BMF* were prognostic biomarkers, we analyzed the non-trastuzumab-treated cohort. *SHB, HAS2* and *BMF* were not identified as beneficial or risk factors in these patients (Fig. S15 d, e and f, Additional file 2). The Kaplan–Meier curves showed a trend towards better overall survival with *HAS2* or *SHB* expression above the optimized threshold in patients not treated with trastuzumab (Fig. S16 a and b, Additional file 2) and no significance using the quantile threshold 50% (Fig. S17 a and b, Additional file 2).

**Table 2:** Patient characteristics and patient treatment groups

Characteristics		All patients (*n* = 100)
**Age**	median (years)	65.5 ± 11.4
**Sex**	male	76
	female	24
**BMI**	median (kg/m^2^)	24.1 ± 3.7
**ECOG**	0–1	88
	> 1	7
**Primary tumor location**	Cardia (AEG I-III)	55
	Non-Cardia	44
**Chemotherapy treatment**	perioperative	29
	w/o perioperative	71
**Primary tumor resection**	yes	48
	no	52
	R0	36
**Grading**	G1-2	41
	G3-4	58
**Number of metastatic sites**	1	64
	> 1	36
**Best response**	Complete Response	6
	Partial Response	20
	Stable Disease	27
	Progressive Disease	30
**Central HER2 IHC score**	0–1	58
	2	16 (11: CISH < 2; 5: CISH ≥ 2)
	3	26
**Central HER2 CISH score**	< 2	61
	≥ 2	32
**Local HER2 IHC score**	0–1	28
	2	8
	3	45
**HER2 targeted treatment**	Trastuzumab	55
	w/o Trastuzumab	45

*AEG* (adenocarcinoma of the esophagogastric junction) according to the Siewert classification, *BMI* (body mass index), *CISH* (chromogenic in situ hybridization), *ECOG* (Eastern Cooperative Oncology Group Performance Status), *IHC* (immunohistochemistry), *R0* (complete resection), *w/o* (without)

## Discussion

### Summary and relevance of the results in the context of previous studies

We have designed a large puzzle to solve the different aspects of HER-targeting in gastric cancer and have used different means to explore these aspects. In previous studies, we have investigated the impact of cetuximab, trastuzumab and afatinib on the activation of the therapeutic targets EGFR and HER2, and whether the effects on membrane receptors affect downstream signalling.

By analysing the effects of treating the MKN1 cell line with epidermal growth factor EGF and cetuximab, it was shown that the MKN1 cell line was sensitive to treatment with cetuximab in various phenotypic assays (proliferation assay, motility assay, invasion assay), while other cell lines studied were either not sensitive at all (Hs746T, LMSU) or only sensitive in certain assays (AGS). Cetuximab inhibited EGFR, MAPK and AKT activity and related components of the EGFR pathway to varying degrees in the cetuximab-sensitive MKN1 cells. In contrast, Hs746T cells did not respond to cetuximab treatment. It can be concluded that the different phenotypic behavior of the cells was linked to their molecular response to treatment. From these results, it can be concluded that components of the EGFR signaling network are important regulators of the phenotypic and molecular response to treatment with cetuximab [16].

We have shown in previous studies that the kinase activity of EGFR and HER2 in the gastric cancer cell lines NCI-N87, MKN1 and MKN7 is selectively inhibited by trastuzumab and afatinib. The efficacy of trastuzumab in the cell lines studied depended on whether HER2 was activated or not. The effects of treatment with trastuzumab monotherapy were not transferred to the intracellular kinase network. In contrast, afatinib alone or in combination with trastuzumab inhibited HER kinases in all cell lines, i.e. the effects of monotherapy and combination therapy were transferred to intracellular kinases. These observations were complemented by phenotypic observation of cell proliferation [17].

A subsequent study compared the effects of cetuximab, trastuzumab and afatinib on intracellular kinase activation and gene expression in gastric cancer cell lines. By analysing intracellular kinase activation, it was shown that cetuximab and afatinib had effects on MAPK3, MEK1, AKT and p70S6K1, although the magnitude of the effects differed between cell lines and treatments. Gene expression analyses led to the identification of important signaling pathways and immune-related signaling pathways that are important for the response to cetuximab and afatinib. Furthermore, cell cycle signaling pathways were identified as important for the response to afatinib. In further experiments, the effects of afatinib on motility were investigated by time-lapse microscopy and on apoptosis by staining with cleaved caspase 3 with the aim of identifying genes involved in the regulation of motility and apoptosis after afatinib treatment. The phenotypic changes were associated with altered biological functions in the Gene Ontology database. The result of these analyses was a list of 14 genes predicted to be potentially involved in the reduction of motility and a list of 44 genes potentially involved in the induction of apoptosis following afatinib treatment. We were able to validate these results by assessing motility parameters after *HBEGF* knockdown [18].

We used the gene expression analysis dataset for functional enrichment analyses as described above [18], and at the same time we also investigated the regulation of gene expression and wanted to explore to what extent gene expression differences are suitable for defining candidate biomarkers. We now present these new findings (both in vitro and clinical) in the current study. Although trastuzumab had less impact on gene expression than cetuximab or afatinib, we were successful in defining *BMF*, *HAS2* and *SHB* as biomarker candidates. Examination of gastric cancer samples from the VARIANZ cohort revealed that *HAS2* and *SHB* are predictive risk factors for response to trastuzumab therapy. The HER family ligands AREG, EREG and HBEGF were identified as biomarker candidates for treatment with cetuximab and afatinib. However, in subsequent experiments, functional validation was only possible for HBEGF. By performing *HBEGF* knockdown experiments when testing drug sensitivity in vitro, we were able to show that HBEGF can be considered a resistance factor for cetuximab treatment.

### Effects of trastuzumab, cetuximab and afatinib on gene expression

Regarding the number of regulated genes, the strongest regulation was observed in NCI-N87 cells after afatinib treatment followed by afatinib-treated MKN7 and MKN1 cells. Smaller numbers of genes were regulated after cetuximab treatment in MKN1 cells. Only slight changes in gene expression were observed after trastuzumab treatment in the responder cell line NCI-N87. Trastuzumab has two modes of action: direct inhibition of HER2 signaling and antibody-dependent cell-mediated cytotoxicity (ADCC) [24, 25]. The discrepancy that trastuzumab is effective in patients but to a lesser extent in cell culture can be explained by the absence of immune cells in vitro.

### Identification of biomarker candidates for trastuzumab, cetuximab and afatinib

*BMF, HAS2* and *SHB* were slightly regulated after trastuzumab treatment in the NCI-N87 responder cell line, hence they are considered as biomarker candidates. Cetuximab and afatinib treatment resulted in regulation of hundreds of genes in the responder cell lines. The most robustly regulated genes had to be selected by comparing different treatments, treatment times or cell lines. Amongst others, *AREG, HBEGF* and *EREG* were identified as biomarker candidates for cetuximab and afatinib treatment.

#### BMF, HAS2 and SHB as candidate biomarkers for trastuzumab treatment

The Bcl-2 modifying factor BMF is involved in apoptotic processes. We showed an increased *BMF* gene expression after trastuzumab, cetuximab and afatinib treatment in gastric cancer cell lines. However, *BMF* gene expression was not confirmed as biomarker for overall survival in trastuzumab-treated or non-trastuzumab-treated gastric cancer patients.

The Hyaluronan Synthase 2 (HAS2) is important for the synthesis of hyaluronan, a core component of the extracellular matrix. We observed a slight downregulation of *HAS2* after trastuzumab, and a strong downregulation after afatinib and cetuximab treatment in gastric cancer cells. Although the *HAS2* reduction following trastuzumab treatment measured by RNA sequencing was not significant, we demonstrated a significant reduction measured by qPCR. Since we hypothesized that a regulation in cultured cells induced by anti-HER therapy indicates the importance of the regulated gene as biomarker, we analyzed *HAS2* gene expression in gastric cancer patients treated with trastuzumab. Indeed, *HAS2* was a predictive biomarker for trastuzumab therapy, as trastuzumab-treated patients with lower *HAS2* expression showed longer overall survival. Yet, this effect was only observed when *HAS2* was measured in pre-therapeutic biopsies and not in resection specimens. This may be explained by the different handling of resection specimens and biopsies in the operating theater. While biopsies go directly into fixation, resections take longer and the stomach mucosa is prone to autodigestion. Thus, we assume that *HAS2* RNA is especially susceptible to degradation. In the non-trastuzumab-treated cohort, the overall survival was not significantly dependent on *HAS2* expression, but patients with higher *HAS2* expression tended to survive longer. In breast cancer patients, higher *HAS2* expression is a poor prognostic factor [26]. Breast cancer cell lines with an aggressive phenotype (MDA-MB-231, HS-578 T) showed higher *HAS2* gene expression and higher hyaluronan levels than cell lines with a less aggressive phenotype. Not only the hyaluronan synthase but also hyaluronan degradation products have been associated with survival. The glycan fragment HexNAc-HexA-HexNAc measured in tumor stroma of gastric cancer FFPE tissues was identified as independent prognostic factor for survival [27].

The Src Homology-2 domain containing protein B (SHB) is an SH2-domain signal protein. Pleiotropic effects of SHB such as regulation of apoptosis, differentiation, proliferation and cytoskeletal alterations were shown in various cell types [28]. Our transcriptome analysis revealed an increase in *SHB* gene expression after 4 h trastuzumab treatment. Analogue to *HAS2*, *SHB* gene expression was analyzed in gastric cancer specimens. *SHB* was a risk factor and trastuzumab-treated patients with lower *SHB* expression showed better overall survival, indicating a potential predictive role for SHB in trastuzumab response. In the non-trastuzumab-treated cohort, patients with higher *SHB* expression tended to survive longer. The limitation that this effect was only observed when *SHB* was measured in biopsies was already discussed in the previous paragraph.

We confirmed our hypothesis that the regulation of a gene after treatment can indicate its importance for response, and thus may be used as biomarker for patient stratification, in case of *HAS2* and *SHB.* However, the validity of this clinical study is limited due to the small sample number. Therefore, a larger patient cohort is required for the independent confirmation of our findings. Due to a rate of *HER2* amplification or HER2 overexpression of 6–30% in gastric cancer patients [29], the recruitment of large patient cohorts with indication for trastuzumab is difficult and long-lasting. In case of the VARIANZ study, 90 of the 514 HER2 characterized patients were identified as HER2 positive [13].

#### The HER-family ligands as candidate biomarkers for cetuximab and afatinib treatment

We identified various candidate biomarkers for cetuximab and afatinib treatment. In this work we focused on the HER-family ligands Amphiregulin (AREG), Heparin Binding EGF Like Growth Factor (HBEGF) and Epiregulin (EREG), since they were regulated by both cetuximab and afatinib treatment in the responder cell lines at both time points. Furthermore, they are of special interest because of their function and their ability to be measured in human plasma.

*AREG, EREG* and *HBEGF* gene expression was reduced after cetuximab and afatinib treatment as demonstrated by RNA sequencing and qPCR. The altered *AREG* expression was additionally confirmed by the level of secreted AREG measured by ELISA. So, in case of *AREG*, the levels of secreted AREG mirrored the gene expression results. In contrast, secreted HBEGF and EREG were below the detection limit although the gene expression was comparable or even higher than AREG gene expression. This discrepancy indicates that either the translation of *EREG* and *HGEBF* mRNA into protein or the shedding of membrane-tethered pro-EREG and pro-HBEGF is lower. Thus, in our cell lines the function of the endogenous membrane-tethered HBEGF is most likely dominant compared to the secreted HBEGF. Similar observations were made in NUGC-3 gastric cancer 3D spheroid cultures. Blocking of membrane-tethered pro-HBEGF with an antibody suppressed cell proliferation and increased caspase activation. In contrast, an antibody against the secreted HBEGF had no influence on cell proliferation [30].

The functional analysis revealed that AREG knockdown or stimulation had no consequences on proliferation or sensitivity towards cetuximab, trastuzumab or afatinib. HBEGF knockdown or stimulation did not affect afatinib sensitivity in MKN1 and NCI-N87 cells or trastuzumab sensitivity in NCI-N87 cells. However, *HBEGF* knockdown increased cetuximab sensitivity, whereas HBEGF stimulation abolished cetuximab sensitivity in MKN1 cells. Since secreted HB-EGF was not measurable, the effect of *HBEGF* knockdown was mediated by membrane-tethered pro-HBEGF. In contrast, the abolished response after stimulation was mediated by soluble HBEGF. We confirmed the previously demonstrated cetuximab resistance of MKN1 cells after HBEGF stimulation [31]. Additionally, we showed increased cetuximab sensitivity after HBEGF knockdown in the present work further supporting the role of HBEGF as biomarker. HNSCC and colorectal cancer cell lines with acquired cetuximab resistance showed, amongst others, upregulated *HBEGF* gene expression. The resistant phenotype of colorectal cancer cells was reversed by *HBEGF* knockdown, whereas resistance was not reversed by treating HNSCC cells with a neutralizing HBEGF antibody [32, 33]. The trastuzumab responder cell lines GSU and H111-TC demonstrated reduced trastuzumab sensitivity after HBEGF, but not AREG stimulation [31]. However, our experiments with NCI-N87 cells demonstrated that HBEGF stimulation did not affect trastuzumab sensitivity. Thus, we conclude that AREG knockdown or stimulation has no functional consequences, and HBEGF can be considered a cetuximab resistance factor. The effect of HBEGF stimulation on trastuzumab sensitivity is most likely cell line-dependent.

## Conclusions

We compared the effects of cetuximab, trastuzumab and afatinib on gene expression in gastric cancer cell lines. Strongest regulation of gene expression was observed after afatinib treatment followed by cetuximab treatment, whereas treatment with trastuzumab had only a limited impact. Although trastuzumab had minor effects on gene expression, we identified *BMF, HAS2* and *SHB* as candidate biomarkers. The gene expression analysis in gastric cancer specimens revealed that *HAS2* and *SHB* were predictive risk factors for trastuzumab therapy response (Table 3). Therefore, we confirmed our initial hypothesis that a gene regulation caused by anti-HER treatment in cell culture indicates that the regulated gene can be an important biomarker. However, our findings should be confirmed in independent patient cohorts.

**Table 3:** Summary of functionally or clinically validated biomarker candidates

Treatment	Candidate biomarkers	Functionally validated biomarker	Clinically validated biomarkers
Afatinib	AREG, EREG, HBEGF	-	-
Cetuximab	AREG, EREG, HBEGF	HBEGF	-
Trastuzumab	BMF, HAS2, SHB	-	HAS2, SHB

Amongst others, the HER-family ligands *AREG, EREG* and *HBEGF* were identified as biomarker candidates for cetuximab and afatinib treatment. *HBEGF* knockdown resulted in enhanced cetuximab sensitivity, whereas stimulation with HBEGF abolished cetuximab response (Table 3). From this functional validation experiments we conclude that HBEGF can be considered as a resistance factor for cetuximab treatment. Clinical validation of HBEGF could not be carried out within the framework of this study, but it would of course be very desirable. Trastuzumab and afatinib sensitivity were not altered following HBEGF knockdown or stimulation. AREG knockdown or stimulation did not change sensitivity towards cetuximab, trastuzumab or afatinib.

In summary, we were able to show that the anti-HER targeted therapeutics investigated differ considerably in their ability to influence gene expression. An in-depth study of the regulated genes – as a pilot study—enabled the identification of biomarker candidates that were subsequently validated functionally and/or clinically to show a first indication of importance for predicting treatment outcome.

## Supplementary Information

**Additional file 1: **

**Additional file 2: **

## Data Availability

The datasets generated and analysed during the current study are available in the GEO repository (Accession GSE141352), https://www.ncbi.nlm.nih.gov/geo/query/acc.cgi?acc=GSE141352.

## Abbreviations

ACTB: β-Actin
ADCC: Antibody-dependent cell-mediated cytotoxicity
AEG: Adenocarcinoma of the esophagogastric junction
Afa: Afatinib
AREG: Amphiregulin
BMF: Bcl-2 modifying factor
BMI: Body mass index
Cet: Cetuximab
CI: Confidence interval
CISH: Chromogenic in situ hybridization
ECOG: Eastern Cooperative Oncology Group Performance Status
DMSO: Dimethylsulfoxid
Epiregulin: EREG
EXPAND: Erbitux (cetuximab) in combination with Xeloda (capecitabine) and cisplatin in advanced esophago-gastric cancer
FDR: False discorey rate, adjusted p-value
FFPE: Formalin-fixed paraffin-embedded
GO-term: Gene ontology term
HBEGF: Heparin Binding EGF Like Growth Factor
HNSCC: Head and neck squamous-cell carcinoma
HR: Hazard ratio
HAS2: Hyaluronan Synthase 2
IHC: Immunohistochemistry
KEGG: Kyoto Encyclopedia of Genes and Genomes
log2FC: Log2-transformed fold-change
R0: Complete resection
SHB: Src Homology-2 domain
ToGA: Trastuzumab for Gastric Cancer
Tra: Trastuzumab
Untr: Untreated
w/o: Without
WST-1: Water soluble tetrazolium

## References

[1] Sung H, Ferlay J, Siegel RL, Laversanne M, Soerjomataram I, Jemal A (2021). Global Cancer Statistics 2020: GLOBOCAN Estimates of Incidence and Mortality Worldwide for 36 Cancers in 185 Countries. CA Cancer J Clin.

[2] Lordick F, Janjigian YY (2016). Clinical impact of tumour biology in the management of gastroesophageal cancer. Nat Rev Clin Oncol.

[3] Bang YJ, Van Cutsem E, Feyereislova A, Chung HC, Shen L, Sawaki A (2010). Trastuzumab in combination with chemotherapy versus chemotherapy alone for treatment of HER2-positive advanced gastric or gastro-oesophageal junction cancer (ToGA): a phase 3, open-label, randomised controlled trial. Lancet.

[4] Lordick F, Kang YK, Chung HC, Salman P, Oh SC, Bodoky G (2013). Capecitabine and cisplatin with or without cetuximab for patients with previously untreated advanced gastric cancer (EXPAND): a randomised, open-label phase 3 trial. Lancet Oncol.

[5] Yonsei University. An Open-label, Multicenter Phase II Study of Afatinib Plus Weekly Taxol as Second Line Treatment for Advanced/Recurrent Gastric and Gastroesophageal Junction Cancer. ClinicalTrialsgov [Internet]. Bethesda: National Library of Medicine (US). Available from https://clinicaltrials.gov/ct2/show/NCT02501603. Accessed 04 December 2021.

[6] Memorial Sloan Kettering Cancer Center. A Phase II Study of Afatinib and Paclitaxel in Patients With Advanced HER2-Positive Trastuzumab Refractory Advanced Esophagogastric Cancer. ClinicalTrialsgov [Internet]. Bethesda: National Library of Medicine (US). Available from https://clinicaltrials.gov/ct2/show/NCT01522768. Accessed 04 December 2021.

[7] Yonsei University. The Master Protocol for Biomarker-Integrated Umbrella Trial in Advanced Gastric Cancer. ClinicalTrialsgov [Internet]. Bethesda: National Library of Medicine (US). Available from https://clinicaltrials.gov/ct2/show/NCT02951091. Accessed 04 December 2021.

[8] Sanchez-Vega F, Hechtman JF, Castel P, Ku GY, Tuvy Y, Won H (2019). EGFR and MET Amplifications Determine Response to HER2 Inhibition in ERBB2-Amplified Esophagogastric Cancer. Cancer Discov.

[9] Kim J, Fox C, Peng S, Pusung M, Pectasides E, Matthee E (2014). Preexisting oncogenic events impact trastuzumab sensitivity in ERBB2-amplified gastroesophageal adenocarcinoma. J Clin Invest.

[10] Kim C, Lee CK, Chon HJ, Kim JH, Park HS, Heo SJ (2017). PTEN loss and level of HER2 amplification is associated with trastuzumab resistance and prognosis in HER2-positive gastric cancer. Oncotarget.

[11] Battaglin F, Naseem M, Puccini A, Lenz HJ (2018). Molecular biomarkers in gastro-esophageal cancer: recent developments, current trends and future directions. Cancer Cell Int.

[12] Haffner I, Schierle, K., Luber, B., Maier, D., Geier, B., Raimundez, E., Hasenauer, J., Kretzschmar, A., Fischer von Weikersthal, L., Ahlborn, M., Riera Knorrenschild, J., Rau, B., Siegler, G., Fuxius, S., Decker, T., Wittekind, C., Lordick, F. Central validation of HER2 in gastric cancer: high heterogeneity in HER2 expression and its impact on survival (Abstract # 0783). International Gastric Cancer Congress; 2019; Prague, Czech Republic.

[13] Haffner I, Schierle, K., Maier, D., Geier, B., Raimundez, E., Hasenauer, J., Luber, B., Kretzschmar, A., Fischer von Weikersthal, L., Ahlborn, M., Riera Knorrenschild, J., Rau, B., Siegler, G., Fuxius, S., Decker, T., Wittekind, C., Lordick, F. HER2positive gastric cancer: intermediate HER2 expression levels are related to high test deviation rates between local and central pathology and worse survival (Abstract # V1069). Jahrestagung der Deutschen, Österreichischen und Schweizerischen Gesellschaften für Hämatologie und Medizinische Onkologie; 2019; Berlin, Germany.

[14] Lordick F, Haffner, I., Luber, B., Maier, D., Raimundez, E., Hasenauer, J., Kretzschmar, A., Fischer von Weikersthal, L., Ahlborn, M., Riera Knorrenschild, J., Siegler, G., Rau, B., Fuxius, S., Decker, T., Schierle, K., Wittekind, C. Heterogeneity of HER2 expression in gastric cancer (GC) leads to high deviation rates between local and central testing and hampers effıcacy of anti-HER2 therapy: Survival results from the VARIANZ study (Abstract # 2615). Annual Meeting of the American Association for Cancer Research; 2018; Illinois, USA.

[15] Haffner I, Schierle K, Raimundez E, Geier B, Maier D, Hasenauer J (2021). HER2 Expression, Test Deviations, and Their Impact on Survival in Metastatic Gastric Cancer: Results From the Prospective Multicenter VARIANZ Study. J Clin Oncol.

[16] Keller S, Kneissl J, Grabher-Meier V, Heindl S, Hasenauer J, Maier D (2017). Evaluation of epidermal growth factor receptor signaling effects in gastric cancer cell lines by detailed motility-focused phenotypic characterization linked with molecular analysis. BMC Cancer.

[17] Keller S, Zwingenberger G, Ebert K, Hasenauer J, Wasmuth J, Maier D (2018). Effects of trastuzumab and afatinib on kinase activity in gastric cancer cell lines. Mol Oncol.

[18] Ebert K, Zwingenberger G, Barbaria E, Keller S, Heck C, Arnold R (2020). Determining the effects of trastuzumab, cetuximab and afatinib by phosphoprotein, gene expression and phenotypic analysis in gastric cancer cell lines. BMC Cancer.

[19] Kneissl J, Keller S, Lorber T, Heindl S, Keller G, Drexler I (2012). Association of amphiregulin with the cetuximab sensitivity of gastric cancer cell lines. Int J Oncol.

[20] Kauraniemi P, Hautaniemi S, Autio R, Astola J, Monni O, Elkahloun A (2004). Effects of Herceptin treatment on global gene expression patterns in HER2-amplified and nonamplified breast cancer cell lines. Oncogene.

[21] Eckstein M, Wirtz RM, Gross-Weege M, Breyer J, Otto W, Stoehr R (2018). mRNA-Expression of KRT5 and KRT20 Defines Distinct Prognostic Subgroups of Muscle-Invasive Urothelial Bladder Cancer Correlating with Histological Variants. International journal of molecular sciences.

[22] Breyer J, Wirtz RM, Otto W, Erben P, Kriegmair MC, Stoehr R (2017). In stage pT1 non-muscle-invasive bladder cancer (NMIBC), high KRT20 and low KRT5 mRNA expression identify the luminal subtype and predict recurrence and survival. Virchows Arch.

[23] Szklarczyk D, Gable AL, Lyon D, Junge A, Wyder S, Huerta-Cepas J (2019). STRING v11: protein-protein association networks with increased coverage, supporting functional discovery in genome-wide experimental datasets. Nucleic Acids Res.

[24] Clynes RA, Towers TL, Presta LG, Ravetch JV (2000). Inhibitory Fc receptors modulate in vivo cytotoxicity against tumor targets. Nat Med.

[25] Kono K, Takahashi A, Ichihara F, Sugai H, Fujii H, Matsumoto Y (2002). Impaired antibody-dependent cellular cytotoxicity mediated by herceptin in patients with gastric cancer. Cancer Res.

[26] Passi A, Vigetti D, Buraschi S, Iozzo RV (2019). Dissecting the role of hyaluronan synthases in the tumor microenvironment. FEBS J.

[27] Kunzke T, Balluff B, Feuchtinger A, Buck A, Langer R, Luber B (2017). Native glycan fragments detected by MALDI-FT-ICR mass spectrometry imaging impact gastric cancer biology and patient outcome. Oncotarget.

[28] Welsh M, Jamalpour M, Zang G, Akerblom B (2016). The role of the Src Homology-2 domain containing protein B (SHB) in beta cells. J Mol Endocrinol.

[29] Apicella M, Corso S, Giordano S (2017). Targeted therapies for gastric cancer: failures and hopes from clinical trials. Oncotarget.

[30] Sato S, Kamada H, Watanabe T, Tsuji I, Fan J (2013). Identification of the cancer cell proliferation and survival functions of proHB-EGF by using an anti-HB-EGF antibody. PLoS One.

[31] Kneissl J, Hartmann A, Pfarr N, Erlmeier F, Lorber T, Keller S (2017). Influence of the HER receptor ligand system on sensitivity to cetuximab and trastuzumab in gastric cancer cell lines. J Cancer Res Clin Oncol.

[32] Kumar SS, Tomita Y, Wrin J, Bruhn M, Swalling A, Mohammed M (2017). High early growth response 1 (EGR1) expression correlates with resistance to anti-EGFR treatment in vitro and with poorer outcome in metastatic colorectal cancer patients treated with cetuximab. Clin Transl Oncol.

[33] Boeckx C, Blockx L, de Beeck KO, Limame R, Camp GV, Peeters M (2015). Establishment and characterization of cetuximab resistant head and neck squamous cell carcinoma cell lines: focus on the contribution of the AP-1 transcription factor. Am J Cancer Res.

