# Supplementary material for: Combining gene expression analysis of gastric cancer cell lines and tumor specimens to identify biomarkers for anti-HER therapies—the role of HAS2, SHB and HBEGF
Source: BMC Cancer. 2022 Mar 9;22:254. doi: 10.1186/s12885-022-09335-4 (PMC8908634; doi:10.1186/s12885-022-09335-4)
Supplement: Supplementary file 1 — Additional file 1: [file 12885_2022_9335_MOESM1_ESM.pdf]

Supplemental Tables

Table S1: Number of plated cells

Experiment	Cell lines	Cell number
RNA extraction	MKN1, MKN7, Hs746T	1.7 x 10^4 cells/cm^2
	NCI-N87	2 x 10^4 cells/cm^2
Transfection with siRNA	MKN1	1.7 x 10^4 cells/cm^2
	NCI-N87	2.2 x 10^4 cells/cm^2
WST-1 proliferation assay	MKN1	6 x 10^3 cells/cm^2
	NCI-N87	12 x 10^3 cells/cm^2

Table S2: Number of down- and upregulated genes in MKN1 cells.
MKN1 cells were treated with afatinib (Afa), trastuzumab (Tra), trastuzumab plus afatinib (Tra_Afa), EGF, cetuximab (Cet) or EGF plus cetuximab (EGF_Cet) for 4 hours (4 h) or 24 hours (24 h). A gene is defined as downregulated or upregulated if the log2FC after treatment is <-1 or >1 and the FDR is <0.05. Downregulated or upregulated refers to the first mentioned treatment in comparison to the second mentioned treatment.

row	MKN1	downregulated	upregulated
**1**	24h_Afa vs. 24h_Tra_Afa	0	0
**2**	24h_Afa vs. 24h_untr	349	392
**3**	24h_Tra vs. 24h_Tra_Afa	372	383
**4**	24h_Tra vs. 24h_untr	0	0
**5**	24h_Tra_Afa vs. 24h_untr	394	386
**6**	4h_Afa vs. 4h_Tra_Afa	0	0
**7**	4h_Afa vs. 4h_untr	121	33
**8**	4h_Tra vs. 4h_Tra_Afa	22	124
**9**	4h_Tra vs. 4h_untr	0	0
**10**	4h_Tra_Afa_4h_untr	125	26
**11**	24h_Cet vs. 24h_EGF_Cet	1525	1127
**12**	24h_Cet vs. 24h_untr	132	119
**13**	24h_EGF vs. 24h_EGF_Cet	5	38
**14**	24h_EGF vs. 24h_untr	630	773
**15**	24h_EGF_Cet vs. 24h_untr	336	209
**16**	4h_Cet vs. 4h_EGF_Cet	351	94
**17**	4h_Cet vs. 4h_untr	71	14
**18**	4h_EGF vs. 4h_EGF_Cet	4	33
**19**	4h_EGF vs. 4h_untr	39	264
**20**	4h_EGF_Cet vs. 4h_untr	3	25

Table S3: Number of down- and upregulated genes in NCI-N87 cells.
NCI-N87 cells were treated with afatinib (Afa), trastuzumab (Tra) or trastuzumab plus afatinib (Tra_Afa) for 4 hours (4 h) or 24 hours (24 h). A genes is defined as downregulated or upregulated if the log2FC after treatment is <-1 or >1 and the FDR is <0.05. Downregulated or upregulated refers to the first mentioned treatment in comparison to the second mentioned treatment.

**row**	NCI-N87	downregulated	upregulated
**1**	24h_Afa vs. 24h_Tra_Afa	0	0
**2**	24h_Afa vs. 24h_untr	4263	1954
**3**	24h_Tra vs. 24h_Tra_Afa	1751	4252
**4**	24h_Tra vs. 24h_untr	0	0
**5**	24h_Tra_Afa vs. 24h_untr	4676	2031
**6**	4h_Afa vs. 4h_Tra_Afa	0	0

**7**	4h_Afa vs. 4h_untr	316	256
**8**	4h_Tra vs. 4h_Tra_Afa	351	403
**9**	4h_Tra vs. 4h_untr	0	0
**10**	4h_Tra_Afa_4h_untr	290	262

Table S4: Number of down- and upregulated genes in MKN7 cells.
MKN7 cells were treated with afatinib (Afa), trastuzumab (Tra) or trastuzumab plus afatinib (Tra_Afa) for 4 hours (4 h) or 24 hours (24 h). A genes is defined as downregulated or upregulated if the log2FC after treatment is <-1 or >1 and the FDR is <0.05. Downregulated or upregulated refers to the first mentioned treatment in comparison to the second mentioned treatment.

**row**	MKN7	downregulated	upregulated
**1**	24h_Afa vs. 24h_Tra_Afa	0	0
**2**	24h_Afa vs. 24h_untr	1695	966
**3**	24h_Tra vs. 24h_Tra_Afa	713	956
**4**	24h_Tra vs. 24h_untr	0	0
**5**	24h_Tra_Afa vs. 24h_untr	1684	990
**6**	4h_Afa vs. 4h_Tra_Afa	0	0
**7**	4h_Afa vs. 4h_untr	405	216
**8**	4h_Tra vs. 4h_Tra_Afa	194	328
**9**	4h_Tra vs. 4h_untr	0	0
**10**	4h_Tra_Afa_4h_untr	429	247

Table S5: Number of differentially expressed genes in untreated cell lines Hs746T, MKN1, NCI-N87 and MKN7.
Gene expression in untreated (untr) cells was compared after 4 hours (4 h) or 24 hours (24 h) culture time. A gene is defined as differentially expressed if the log2FC is <-1 or >1 and the FDR is <0.05. Lower or higher expression refers to the first mentioned cell line in comparison to the second mentioned cell line.

	Lower expression	Higher expression
Hs746T_4h_untr vs. MKN1_4h_untr	4013	6227
Hs746T_4h_untr vs. MKN7_4h_untr	4319	4770
Hs746T_4h_untr vs. NCI-N87_4h_untr	4898	5012
MKN1_4h_untr vs. NCI-N87_4h_untr	5980	3786
MKN1_4h_untr vs. MKN7_4h_untr	5184	3394
MKN7_4h_untr vs. NCI-N87_4h_untr	4510	4216
Hs746T_24h_untr vs. MKN1_24h_untr	3840	7258
Hs746T_24h_untr vs. MKN7_24h_untr	4082	5324
Hs746T_24h_untr vs. NCI-N87_24h_untr	4696	5195
MKN1_24h_untr vs. NCI-N87_24h_untr	7027	3784
MKN1_24h_untr vs. MKN7_24h_untr	5771	3644
MKN7_24h_untr vs. NCI-N87_24h_untr	4930	4079

Table S6: Regulated genes after 4 h and 24 h cetuximab and EGF treatment in MKN1 cells.

	MKN1_4h_EGF vs. MKN1_4h_untr		MKN1_24h_EGF vs. MKN1_24h_untr		MKN1_4h_Cet vs. MKN1_4h_untr		MKN1_24h_Cet vs. MKN1 24h_untr	
Gene Symbol	log2FC	FDR	log2FC	FDR	log2FC	FDR	log2FC	FDR
SPRY4	1.6	1.95E-04	2.0	3.40E-07	-2.8	4.51E-11	-2.8	8.35E-11
FOSL1	1.2	2.77E-03	2.1	2.87E-10	-1.8	1.45E-06	-2.4	8.35E-11
DUSP6	1.9	8.26E-05	1.9	4.78E-06	-2.5	1.56E-07	-3.0	3.95E-10
MAFF	1.1	4.89E-05	1.4	2.41E-09	-1.5	2.33E-08	-1.6	3.95E-10

AC018629.1	1.1	1.77E-02	2.8	3.99E-13	-1.9	1.39E-05	-2.5	5.57E-09
PHLDA1	1.5	5.86E-06	1.7	1.77E-08	-2.2	1.20E-10	-2.0	8.94E-09
ERRFI1	1.8	7.03E-13	1.1	1.94E-06	-1.3	2.46E-06	-1.5	2.88E-08
MFSD2A	1.3	3.94E-09	1.6	1.40E-13	-1.6	1.82E-11	-1.4	4.02E-08
TNS4	1.2	5.62E-03	2.7	3.85E-14	-2.0	3.54E-07	-2.1	6.29E-08
EGR2	1.9	5.77E-04	-1.4	3.77E-03	-1.9	1.64E-02	-2.7	4.24E-07
SGK1	1.5	5.66E-08	1.3	1.01E-06	-1.7	1.51E-09	-1.5	4.98E-07
AREG	2.3	9.12E-06	2.0	1.68E-05	-2.0	4.09E-04	-2.6	9.93E-07
HMGA2	1.4	2.02E-09	2.5	4.54E-29	-1.5	7.73E-10	-1.2	3.27E-06
EREG	1.4	7.29E-03	2.0	2.65E-06	-1.9	1.40E-04	-2.2	4.38E-06
PTHLH	2.3	2.50E-23	2.6	3.50E-30	-1.3	1.52E-07	-1.0	5.47E-05
MIR100HG	1.6	4.21E-19	3.1	5.40E-58	-1.5	1.82E-11	-1.1	6.71E-05
IL11	1.5	6.54E-09	2.3	3.12E-19	-1.4	3.92E-06	-1.2	8.33E-05
MYC	1.3	7.35E-06	1.6	7.68E-09	-1.4	4.52E-06	-1.2	1.89E-04
PRR9	2.1	2.11E-06	4.4	1.28E-21	-1.4	1.93E-02	-2.3	8.12E-04
CSF2	2.7	5.42E-11	3.1	8.41E-12	-1.9	1.66E-04	-1.7	9.31E-03
IL8	2.0	2.58E-09	1.9	3.91E-08	-1.8	4.12E-07	-1.1	2.03E-02
RGS4	2.8	3.87E-14	3.5	1.26E-19	-1.7	2.20E-04	-1.1	2.58E-02

Table S7: Regulated genes after 4 h and 24 h afatinib treatment in NCI-N87 and MKN1 cells.

	NCI-N87_4h_Afa vs. NCI-N87_4h_untr		NCI-N87_24h_Afa vs. NCI-N87_24h_untr		MKN1_4h_Afa vs. MKN1_4h_untr		MKN1_24h_Afa vs. MKN1_24h_untr	
**Gene Symbol**	**log2FC**	**FDR**	**log2FC**	**FDR**	**log2FC**	**FDR**	**log2FC**	**FDR**
BMF	3.5	3.2E-126	4.2	5.7E-184	1.5	3.0E-04	1.8	6.6E-07
F3	-2.7	3.7E-85	-3.9	5.2E-161	-1.8	1.3E-11	-1.8	5.3E-12
SPRY4	-4.0	4.6E-79	-6.4	2.9E-144	-3.8	2.9E-19	-4.7	1.7E-26
SPRED2	-1.4	4.2E-37	-2.7	2.8E-126	-1.2	4.3E-13	-1.6	6.3E-22
TNS4	-2.9	1.7E-58	-4.4	2.3E-123	-2.5	4.8E-11	-2.8	2.8E-14
SPRED1	-1.3	1.7E-23	-2.7	1.1E-92	-1.1	1.2E-07	-1.8	6.0E-19
FOSL1	-3.1	3.1E-46	-4.6	1.2E-89	-2.3	2.4E-11	-2.8	5.7E-16
EPHA2	-2.7	1.5E-67	-3.1	2.3E-88	-1.7	1.9E-17	-1.9	9.0E-22
SLC20A1	-1.6	4.2E-37	-2.3	5.6E-78	-1.5	1.1E-06	-1.2	1.1E-05
PLK3	-2.5	1.7E-59	-2.9	8.0E-78	-1.1	1.4E-10	-1.0	4.2E-09
ENC1	-1.6	1.1E-35	-2.4	1.9E-76	-1.4	8.3E-14	-1.6	2.0E-18
EREG	-1.9	1.1E-27	-3.3	5.5E-72	-2.3	1.6E-06	-2.9	9.0E-11
DUSP7	-1.5	2.7E-26	-2.4	7.9E-68	-1.1	2.2E-08	-1.1	2.7E-09
DUSP4	-3.4	4.3E-44	-4.2	1.3E-63	-2.5	1.6E-18	-3.2	4.7E-27
LIF	-2.1	2.8E-39	-2.6	2.4E-60	-1.7	1.4E-18	-1.1	3.8E-08
ETV5	-1.4	1.0E-06	-4.8	3.4E-60	-1.3	2.1E-07	-2.9	8.4E-32
AC018629.1	-2.0	9.6E-20	-3.6	7.0E-60	-2.2	1.5E-07	-3.3	1.4E-15
ERRFI1	-1.5	8.4E-30	-2.1	3.3E-56	-1.6	1.3E-09	-1.9	5.5E-15
ADORA2B	-1.6	1.1E-15	-3.2	7.8E-56	-1.0	2.2E-11	-1.3	1.1E-18
DUSP6	-4.9	2.4E-35	-5.8	4.2E-48	-3.4	7.0E-14	-4.5	4.4E-21

HBEGF	-1.3	9.4E-13	-2.6	3.1E-47	-1.2	9.3E-03	-2.1	1.2E-08
CBARP	-1.4	9.6E-11	-3.1	3.6E-45	-1.1	7.6E-12	-1.5	1.1E-19
AREG	-1.7	2.7E-18	-2.7	9.7E-44	-2.4	3.5E-06	-3.5	8.6E-12
MYC	-2.2	8.8E-33	-2.4	3.6E-39	-1.9	5.8E-11	-1.5	1.8E-07
MAFF	-1.8	1.1E-24	-2.2	2.7E-37	-1.9	1.4E-14	-2.3	6.4E-20
PRAG1	-1.3	6.9E-18	-1.9	6.3E-37	-1.3	4.8E-14	-1.1	2.8E-09
SPRY2	-1.5	2.9E-12	-2.6	2.1E-35	-1.4	3.1E-15	-1.4	7.3E-15
STON1-GTF2A1L	1.8	3.3E-06	4.0	3.6E-34	1.6	2.4E-02	1.4	6.0E-03
AP005233.2	-3.0	4.9E-27	-3.2	5.1E-32	-1.4	2.4E-05	-3.0	5.2E-21
IL8	-4.1	6.0E-23	-5.0	2.5E-31	-2.1	2.9E-09	-1.5	3.1E-04
AC027117.2	1.3	5.3E-11	2.1	1.7E-30	1.3	2.4E-02	1.1	2.0E-03
PHLDA1	-2.1	1.6E-25	-2.2	4.5E-30	-2.9	9.1E-19	-2.6	2.6E-16
AL590560.1	1.0	5.2E-07	1.9	2.5E-28	1.3	2.6E-03	1.1	2.9E-04
SPRY4-AS1	-1.4	1.0E-03	-4.6	5.8E-28	-1.4	1.1E-02	-3.1	2.3E-11
MFSD2A	-2.0	9.1E-16	-2.6	9.7E-28	-1.9	3.9E-17	-1.9	1.0E-15
DUSP5	-2.6	2.5E-17	-3.1	1.6E-24	-1.5	7.6E-06	-1.7	1.0E-08
EGR1	-4.7	1.6E-15	-5.5	2.8E-21	-3.7	1.5E-18	-5.0	1.2E-30
GPR3	-2.1	3.8E-12	-3.1	4.3E-21	-2.3	3.4E-16	-2.0	4.9E-11
CSF2	-3.9	1.0E-19	-4.8	2.7E-19	-2.4	2.0E-07	-1.6	7.1E-03
AC004585.1	-2.4	3.9E-10	-3.3	1.4E-18	-1.8	4.7E-08	-2.6	2.2E-13
FOS	-3.2	1.6E-10	-4.1	5.7E-17	-1.4	9.4E-08	-1.3	8.0E-08
IER3	-1.3	1.8E-06	-2.0	1.6E-15	-1.8	2.2E-06	-2.0	4.4E-08
ANKRD1	-2.7	5.4E-17	-1.1	6.1E-12	-1.8	1.0E-04	-1.4	9.9E-04
IL1	-1.9	9.4E-03	-2.3	1.1E-04	-2.0	4.3E-05	-1.2	2.0E-02
LINC01629	-1.9	9.9E-04	-2.0	1.9E-04	-1.4	1.6E-02	-2.8	1.4E-06

Table S8: Correlation of gene expression/protein secretion measured by RNA sequencing, qPCR and ELISA.

		RNA Seq /qPCR		RNA Seq/qPCR/ELISA	
Gene	Cell line	Pearson	p adjusted	Pearson	p adjusted
AREG	MKN1	0.9877	0.0006	0.8252	0.054
AREG	MKN7	0.9999	0.0186	0.9989	0.054
AREG	Hs746T	0.4767	0.1922	0.2858	0.3644
AREG	NCI-N87	0.9936	0.0509	0.9819	0.1011
EREG	MKN1	0.9886	0.0006		
EREG	MKN7	1.0000	0.0186		
EREG	Hs746T	-0.8841	0.9903		
EREG	NCI-N87	1.0000	0.0186		
BMF	MKN1	0.9995	3.14E-06		
BMF	NCI-N87	0.9992	0.0371		
SHB	NCI-N87	0.9999	0.0186		
