# Supplementary material for: Combining gene expression analysis of gastric cancer cell lines and tumor specimens to identify biomarkers for anti-HER therapies—the role of HAS2, SHB and HBEGF
Source: BMC Cancer. 2022 Mar 9;22:254. doi: 10.1186/s12885-022-09335-4 (PMC8908634; doi:10.1186/s12885-022-09335-4)
Supplement: Supplementary file 2 — Additional file 2: [file 12885_2022_9335_MOESM2_ESM.pdf]

Figure S1

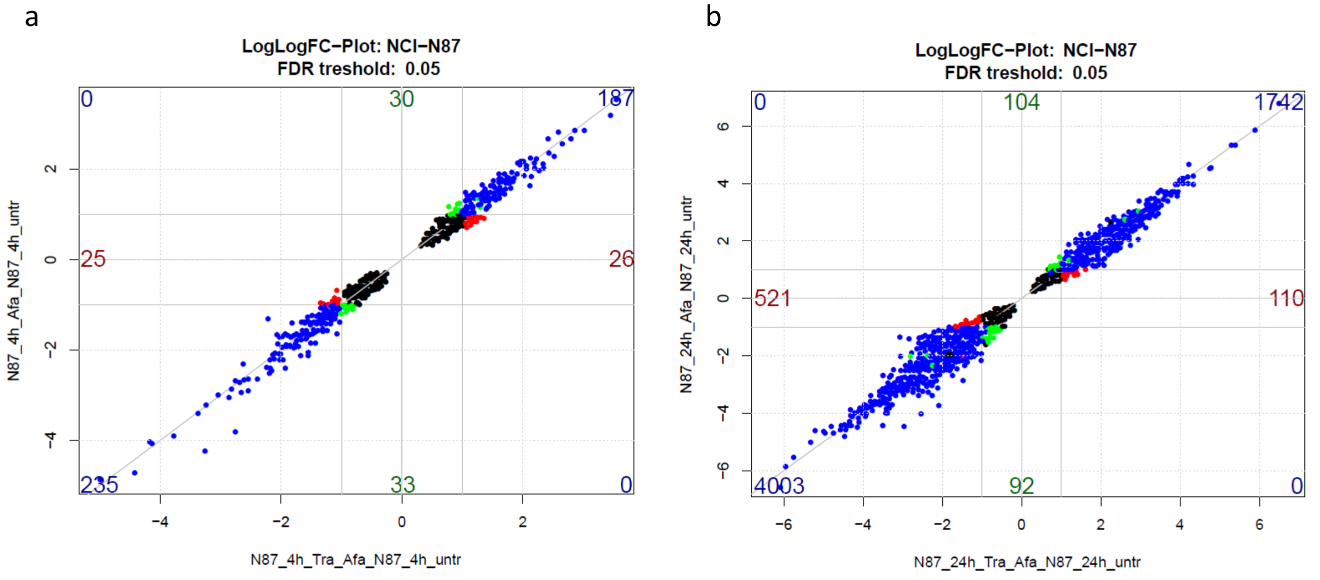

Figure S1: Comparison of regulated genes after afatinib and trastuzumab plus afatinib treatment in NCI-N87 cells.
The differentially expressed genes after 4 h (a) or 24 h (b) afatinib (Afa) or trastuzumab plus afatinib (Tra+Afa) treatment were selected (log2FC<-1 or >1, FDR<0.05) and compared using R. 26 (4 h) or 110 (24 h) genes were upregulated by Tra+Afa and not regulated by Afa. 25 (4 h) or 521 (24 h) genes were downregulated by Tra+Afa and not regulated by Afa. LogLogFC-Plot stands for scatter plot.

Figure S2

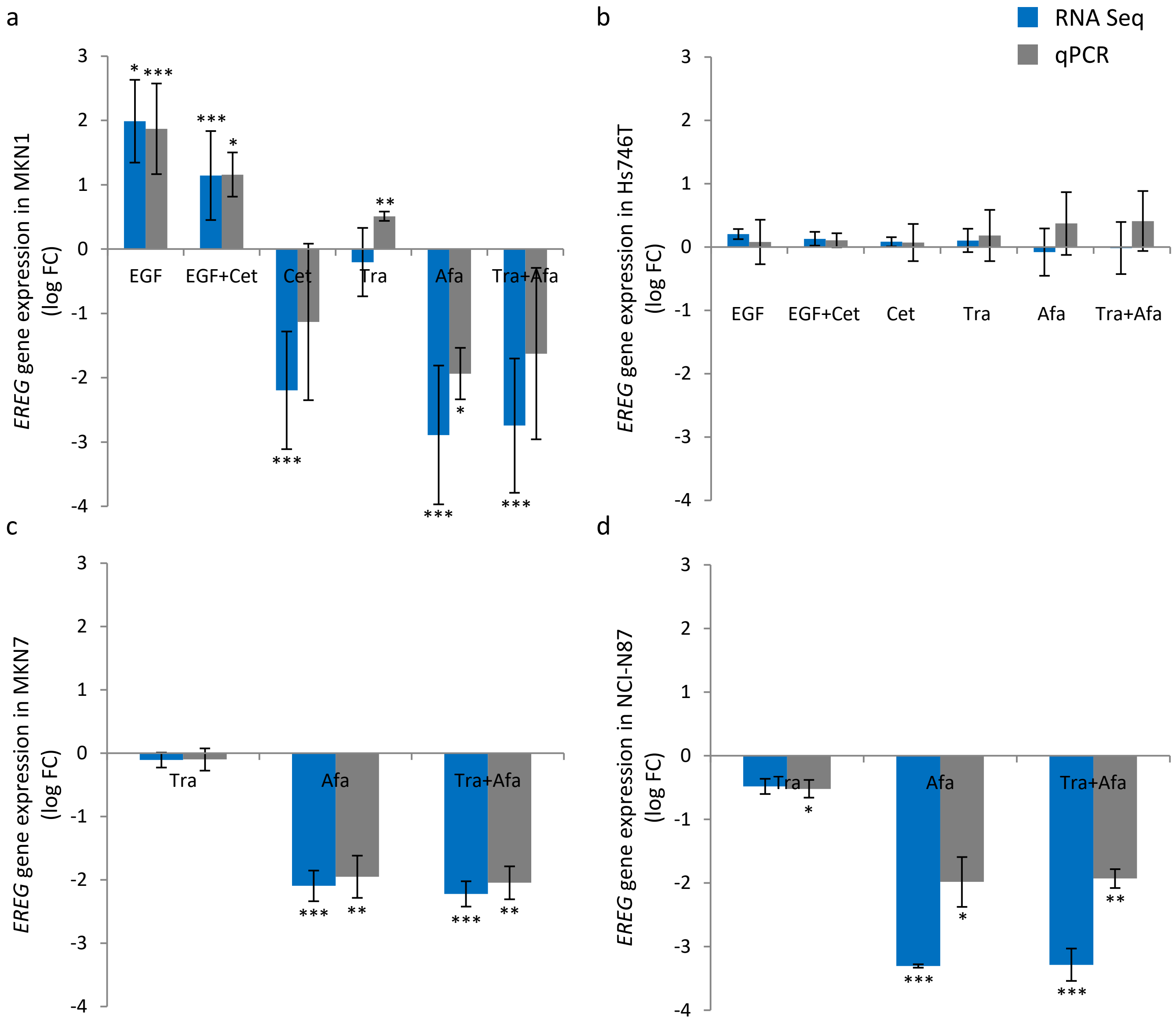

Figure S2: *EREG* gene expression measured by RNA Sequencing and qPCR.

MKN1 (a) and Hs746T (b) cells were treated with EGF, EGF plus cetuximab (EGF+Cet), cetuximab (Cet), trastuzumab (Tra), afatinib (Afa) or trastuzumab plus afatinib (Tra+Afa) for 24 h. MKN7 (c) and NCI-N87 (d) were treated with trastuzumab (Tra), afatinib (Afa) or trastuzumab plus afatinib (Tra+Afa) for 24 h. *EREG* gene expression was measured by RNA Sequencing and qPCR. The mean of three biological experiments with standard deviation is shown. Statistically significant effects compared to untreated are indicated by *$p<0.05$, **$p<0.01$ or ***$p<0.001$ (one-sample t-test).

Figure S3

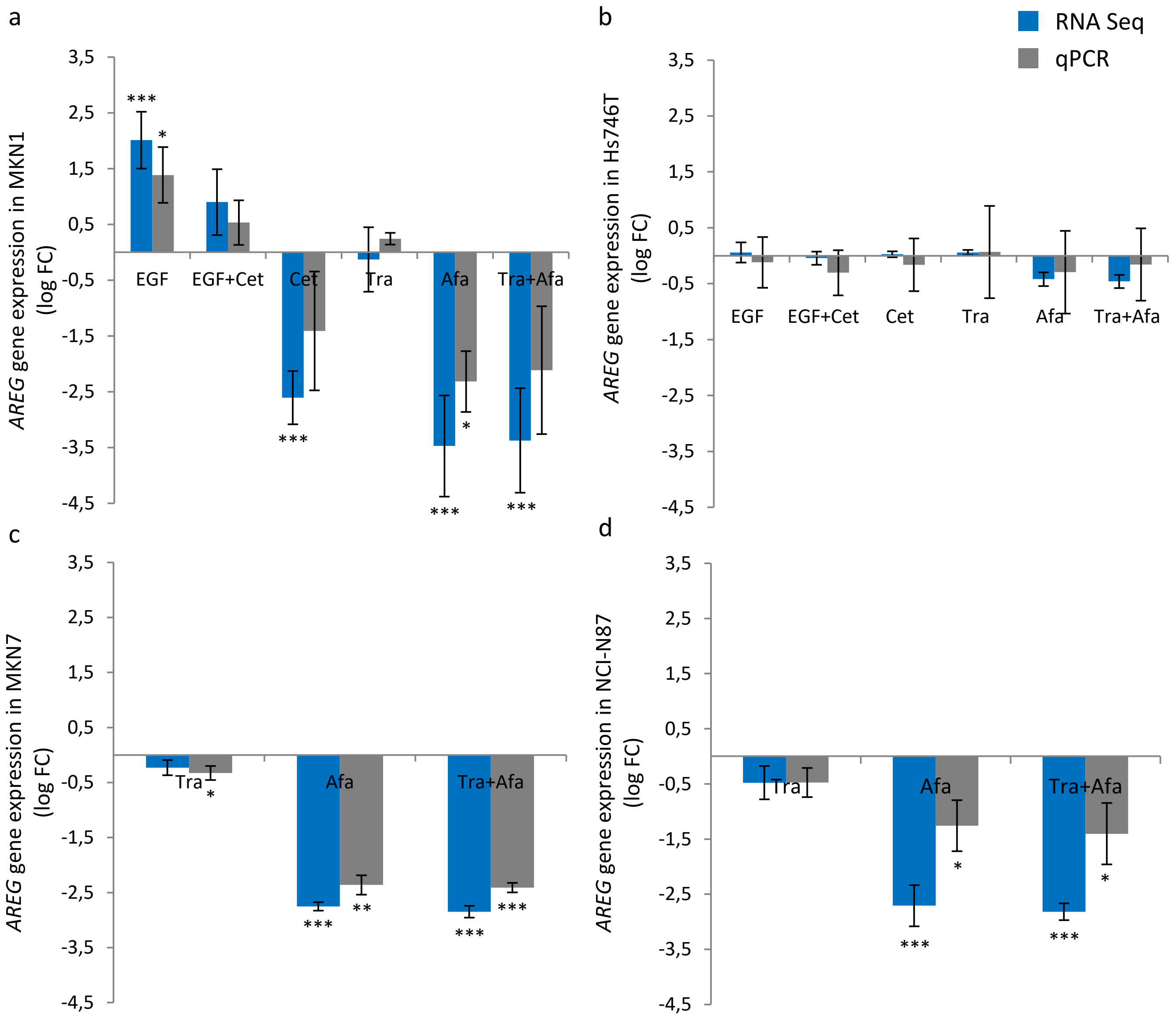

Figure S3 ***AREG*** **gene expression measured by RNA Sequencing and qPCR.**
MKN1 (a) and Hs746T (b) cells were treated with EGF, EGF plus cetuximab (EGF+Cet), cetuximab (Cet), trastuzumab (Tra), afatinib (Afa) or trastuzumab plus afatinib (Tra+Afa) for 24 h. MKN7 (c) and NCI-N87 (d) were treated with trastuzumab (Tra), afatinib (Afa) or trastuzumab plus afatinib (Tra+Afa) for 24 h. *AREG* gene expression was measured by RNA Sequencing and qPCR. The mean of three biological experiments with standard deviation is shown. Statistically significant effects compared to untreated are indicated by *p<0.05, **p<0.01 or ***p<0.001 (one-sample t-test).

Figure S4

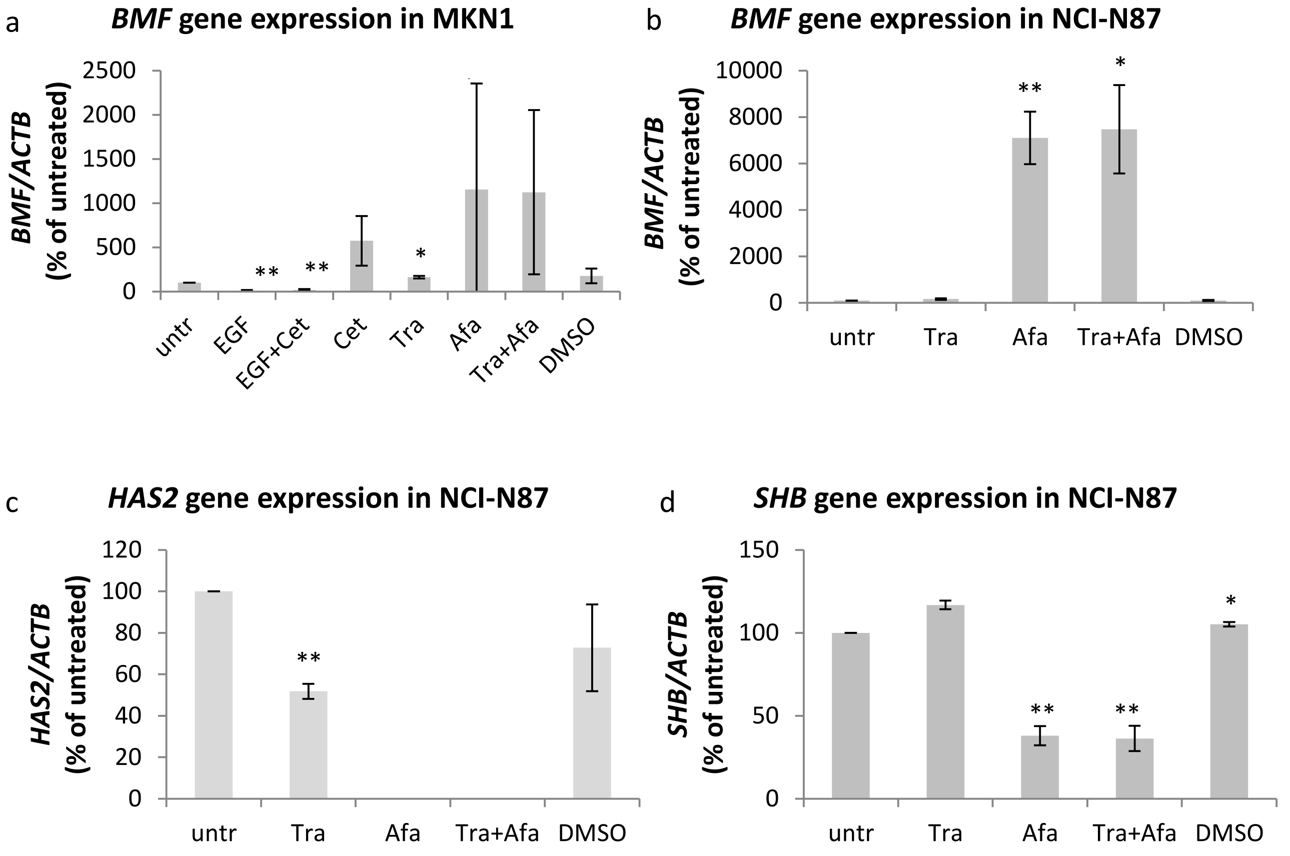

Figure S4: *BMF, HAS2*, and *SHB* gene expression measured by qPCR.
MKN1 (a) cells were treated with EGF, EGF plus cetuximab (EGF+Cet), cetuximab (Cet), trastuzumab (Tra), afatinib (Afa), trastuzumab plus afatinib (Tra+Afa) or DMSO. NCI-N87 (b, c, d) were treated with trastuzumab (Tra), afatinib (Afa), trastuzumab plus afatinib (Tra+Afa) or DMSO. The selected treatment times were 24 h for *BMF* (a, b) and *HAS2* (c) and 4 h for SHB (d). *BMF* (a, b), *HAS2* (c) and *SHB* (d) gene expression was measured by RNA Sequencing and qPCR. The mean of three biological experiments with standard deviation is shown. Statistically significant effects compared to untreated are indicated by *p<0.05 or **p<0.01 (one-sample t-test).

Figure S5

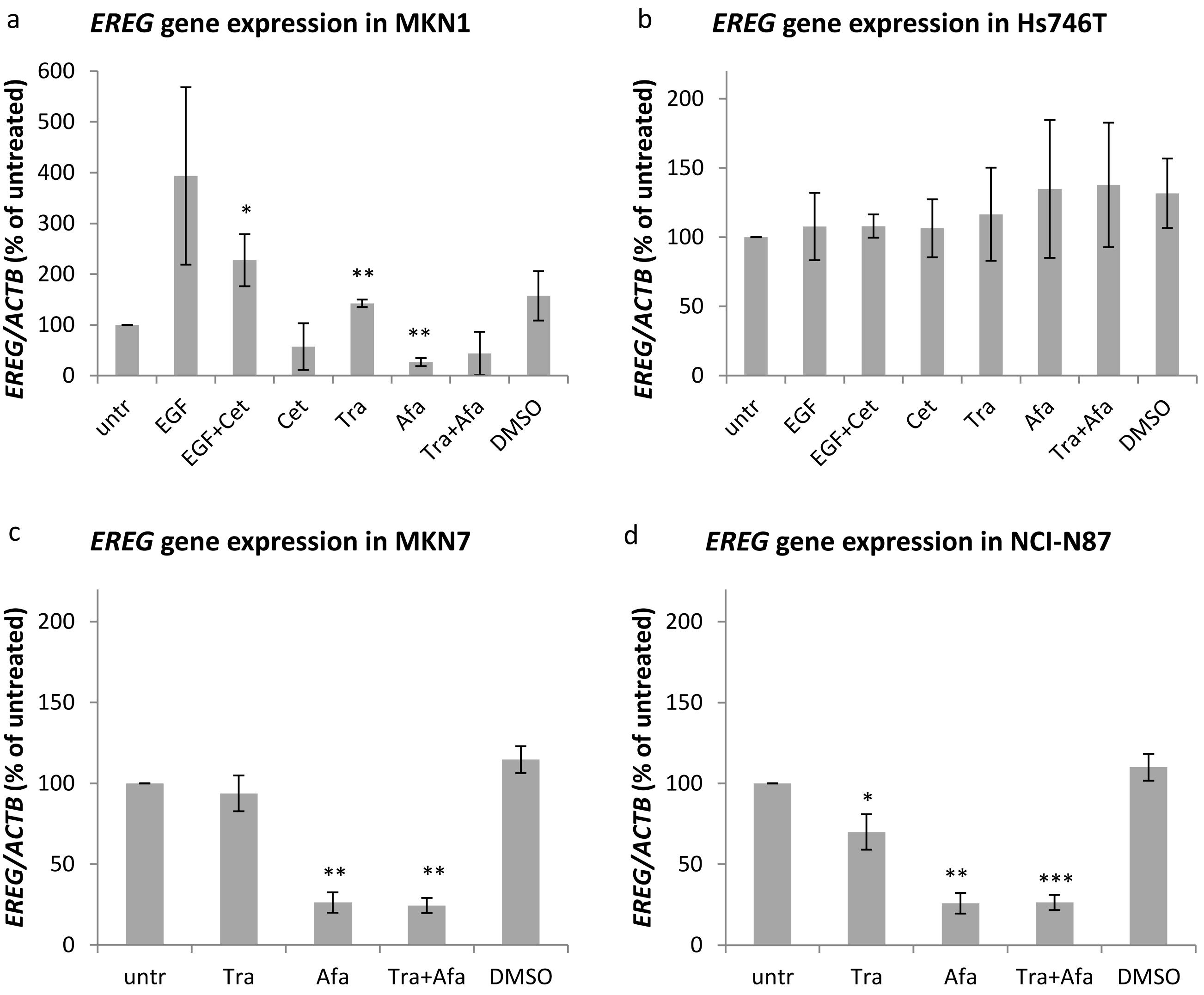

Figure S5: *EREG* gene expression measured by qPCR.
MKN1 (a) and Hs746T (b) cells were treated with EGF, EGF plus cetuximab (EGF+Cet), cetuximab (Cet), trastuzumab (Tra), afatinib (Afa), trastuzumab plus afatinib (Tra+Afa) or DMSO for 24 h. MKN7 (c) and NCI-N87 (d) were treated with trastuzumab (Tra), afatinib (Afa), trastuzumab plus afatinib (Tra+Afa) or DMSO for 24 h. *EREG* gene expression was measured by RNA Sequencing and qPCR. The mean of three biological experiments with standard deviation is shown. Statistically significant effects compared to untreated are indicated by *p<0.05, **p<0.01 or ***p<0.001 (one-sample t-test).

Figure S6

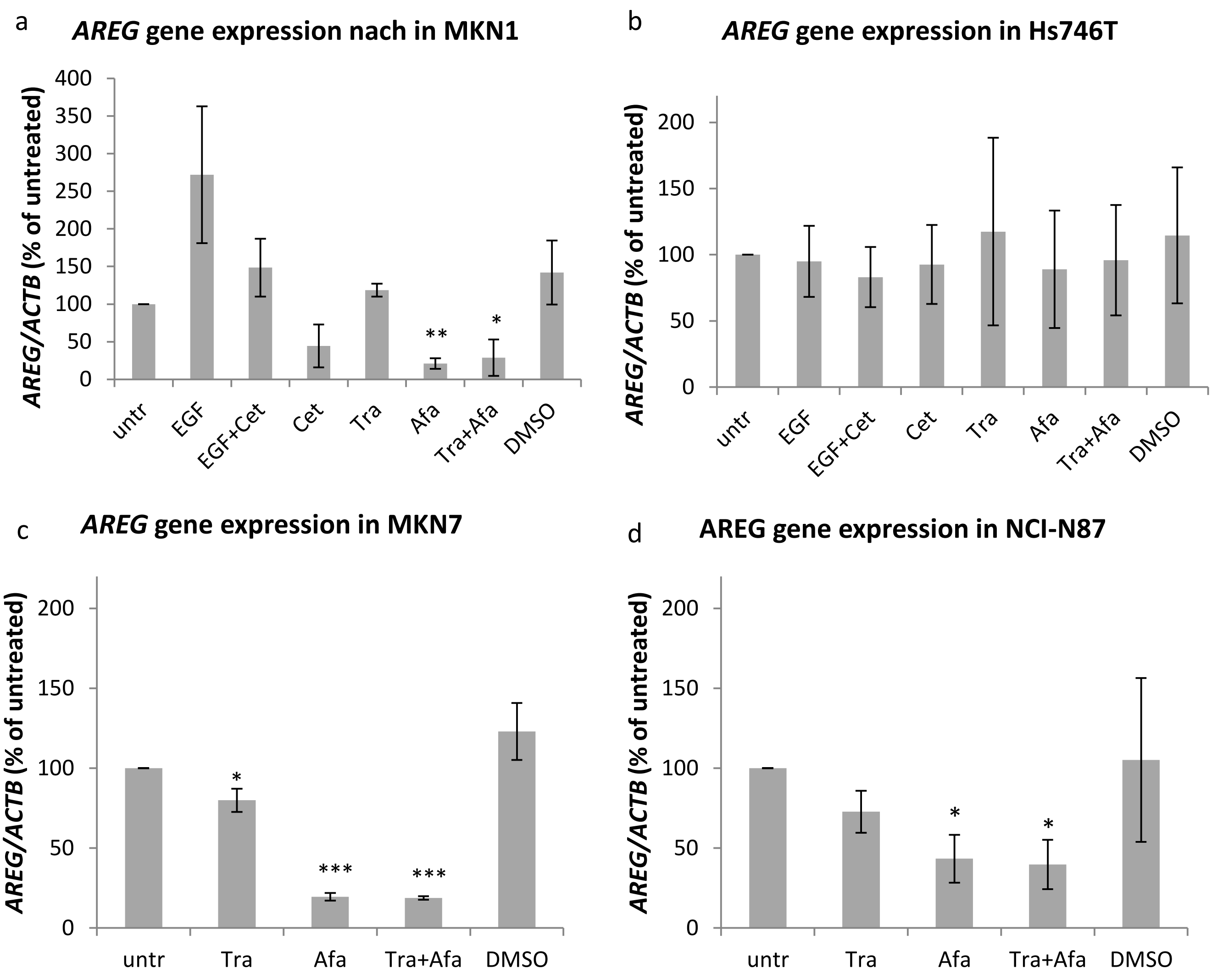

Figure S6: AREG gene expression measured by qPCR.

MKN1 (a) and Hs746T (b) cells were treated with EGF, EGF plus cetuximab (EGF+Cet), cetuximab (Cet), trastuzumab (Tra), afatinib (Afa), trastuzumab plus afatinib (Tra+Afa) or DMSO for 24 h. MKN7 (c) and NCI-N87 (d) were treated with trastuzumab (Tra), afatinib (Afa), trastuzumab plus afatinib (Tra+Afa) or DMSO for 24 h. *AREG* gene expression was measured by RNA Sequencing and qPCR. The mean of three biological experiments with standard deviation is shown. Statistically significant effects compared to untreated are indicated by *p<0.05, **p<0.01 or ***p<0.001 (one-sample t-test).

Figure S7

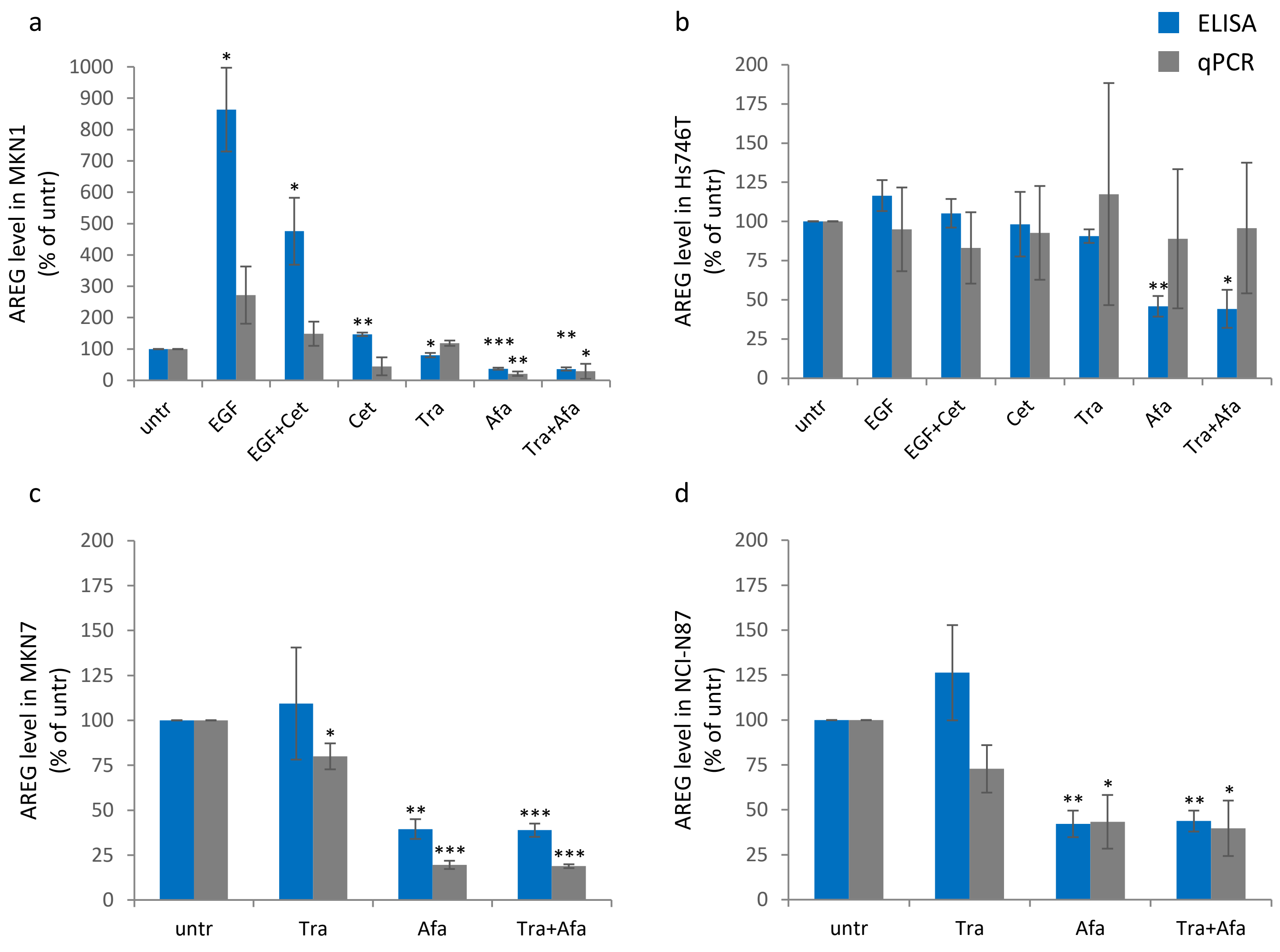

Figure S7: AREG secretion and *AREG* gene expression measured by ELISA and qPCR.
MKN1 (a) and Hs746T (b) cells were treated with EGF, EGF plus cetuximab (EGF+Cet), cetuximab (Cet), trastuzumab (Tra), afatinib (Afa) or trastuzumab plus afatinib (Tra+Afa) for 24 h. MKN7 (c) and NCI-N87 (d) were treated with trastuzumab (Tra), afatinib (Afa) or trastuzumab plus afatinib (Tra+Afa) for 24 h. *AREG* gene expression was measured by qPCR and secreted AREG was measured by ELISA. The mean of three biological experiments with standard deviation is shown. Statistically significant effects compared to untreated are indicated by *$p<0.05$, **$p<0.01$ or ***$p<0.001$ (one-sample t-test).

Figure S8

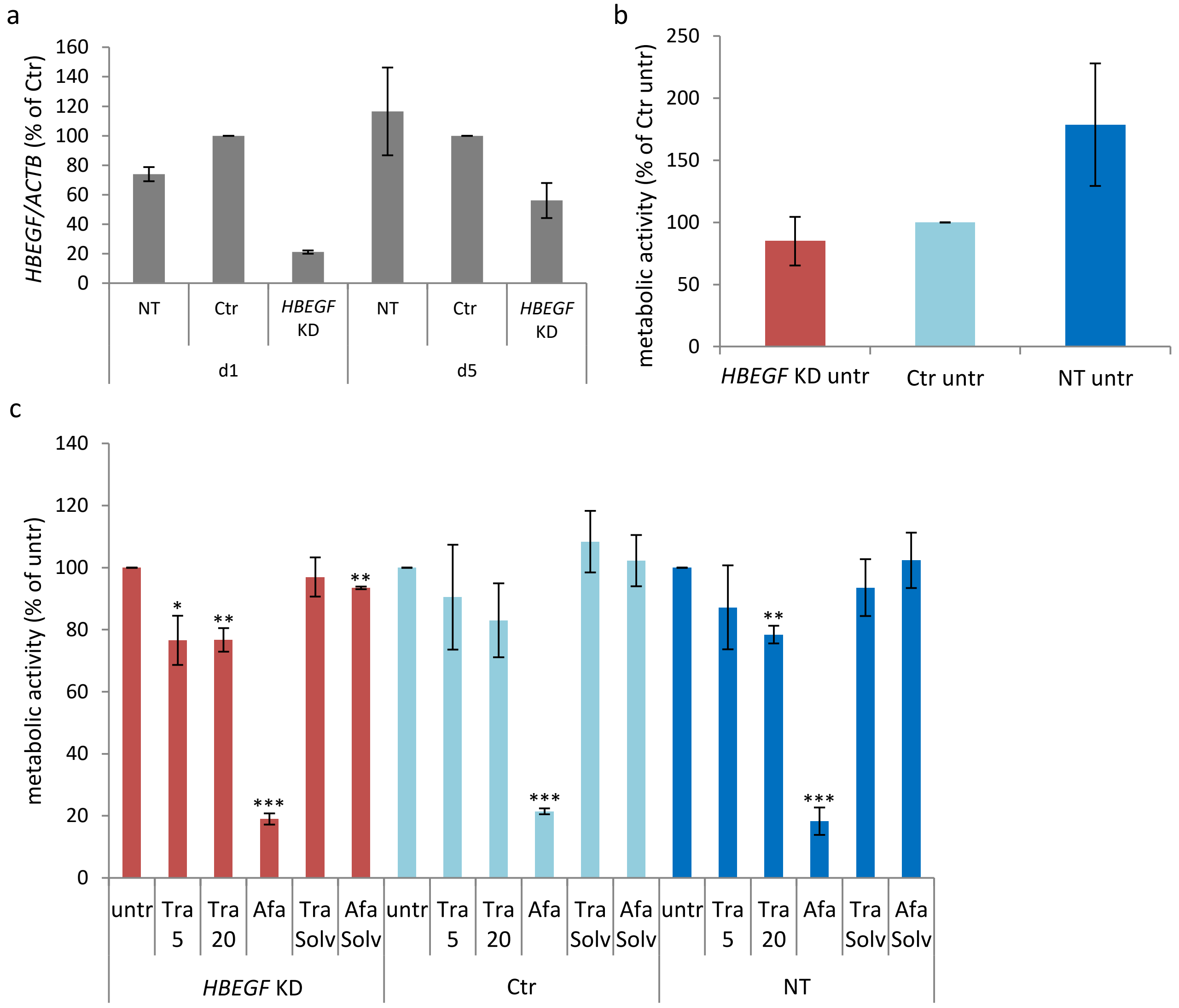

Figure S8: Effects of trastuzumab and afatinib on proliferation after *HBEGF* knockdown in NCI-N87 cells.
NCI-N87 cells were transfected with negative-control (Ctr) or *HBEGF* (*HBEGF* KD) siRNA. Non-transfected (NT cells) were used as control. The knockdown was checked on RNA level on day 1 (d1) and day 5 (d5) after transfection (a). The metabolic activity was measured by WST-1 proliferation assay for 72 h in the untreated (b) and treated (c) state. Cells were treated with 5 µg/ml trastuzumab (Tra 5), 20 µg/ml trastuzumab (Tra 20), 0.5 µM afatinib (Afa) or the corresponding solvents (Tra Solv, Afa Solv) for 72 h. Shown are the mean values from three experiments with standard deviation. Significant effects compared to untreated within a group (*HBEGF* KD, Ctr, NT) are indicated by *p<0.05, ** p<0.01 or ***p<0.001 (one-sample t-test). Significant effects compared to Ctr or NT cells with the same treatment were not observed (two-sample t-test).

Figure S9

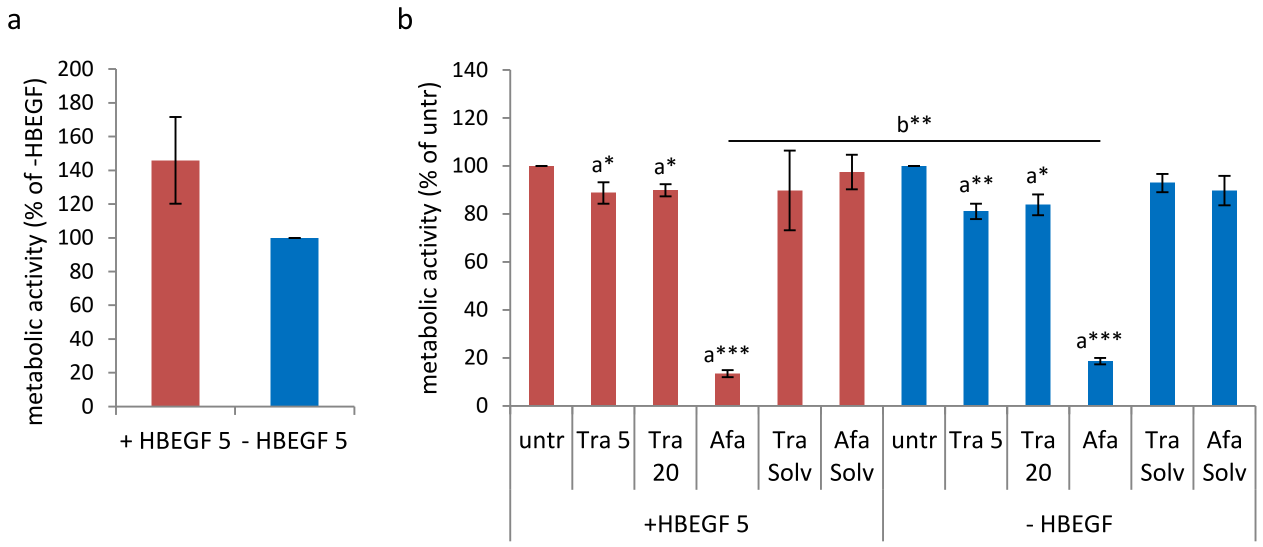

Figure S9: Effects of trastuzumab and afatinib on proliferation after HBEGF stimulation in NCI-N87 cells.
NCI-N87 cells were stimulated with 5 ng/ml HBEGF (+HBEGF 5) or not stimulated (-HBEGF 5). The metabolic activity was measured by WST-1 proliferation assay for 72 h in the untreated (a) and treated (b) state. Cells were treated with 5 µg/ml trastuzumab (Tra 5), 20 µg/ml Trastuzumab (Tra 20), 0.5 µM afatinib (Afa) or the appropriate solvents (Tra Solv, Afa Solv) for 72 h. Shown are the mean values from three experiments with standard deviation. Significant effects compared to untreated within a group (+HBEGF 5, -HBEGF 5) are indicated by a*p<0.05, a**p<0.01 or a***p<0.001 (one-sample sample t-test). Significant effects compared to -HBEGF 5 with the same treatment are indicated by b*p<0.05 (two-sample t-test).

Figure S10

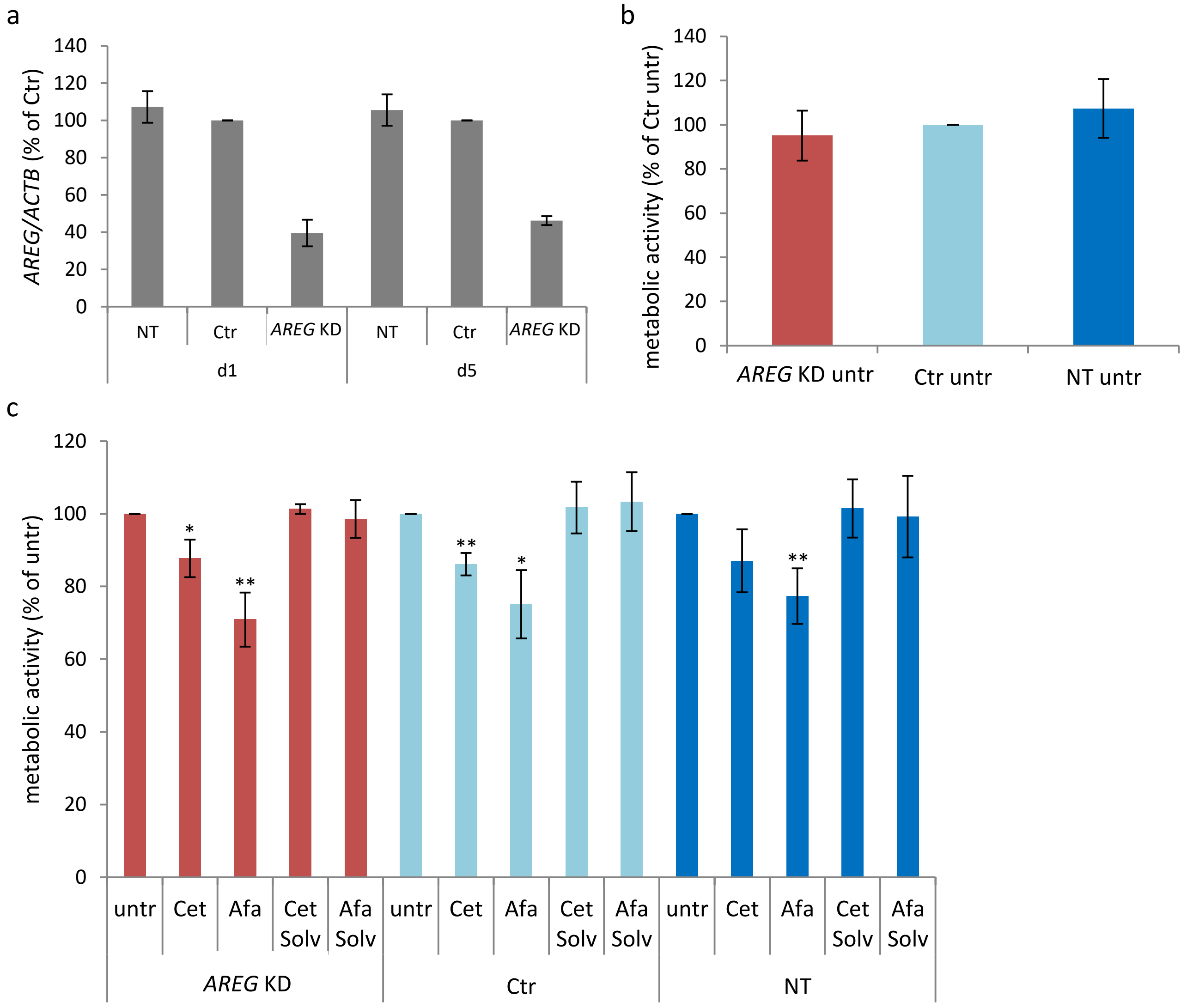

Figure S10: Effects of cetuximab and afatinib on proliferation after *AREG* knockdown in MKN1 cells.
MKN1 cells were transfected with negative-control (Ctr) or *AREG* (*AREG* KD) siRNA. Non-transfected (NT cells) were used as control. The knockdown was checked on RNA level on day 1 (d1) and day 5 (d5) after transfection (a). The metabolic activity was measured by WST-1 proliferation assay for 72 h in the untreated (b) and treated (c) state. Cells were treated with 1 µg/ml cetuximab (Cet 1), 10 µg/ml cetuximab (Cet 10), 0.5 µM afatinib (Afa) or the corresponding solvents (Cet Solv, Afa Solv) for 72 h. Shown are the mean values from three experiments with standard deviation. Significant effects compared to untreated within a group (*AREG* KD, Ctr, NT) are indicated by *p<0.05, ** p<0.01 or ***p<0.001 (one-sample t-test). Significant effects compared to Ctr or NT cells with the same treatment were not observed (two-sample t-test).

Figure S11

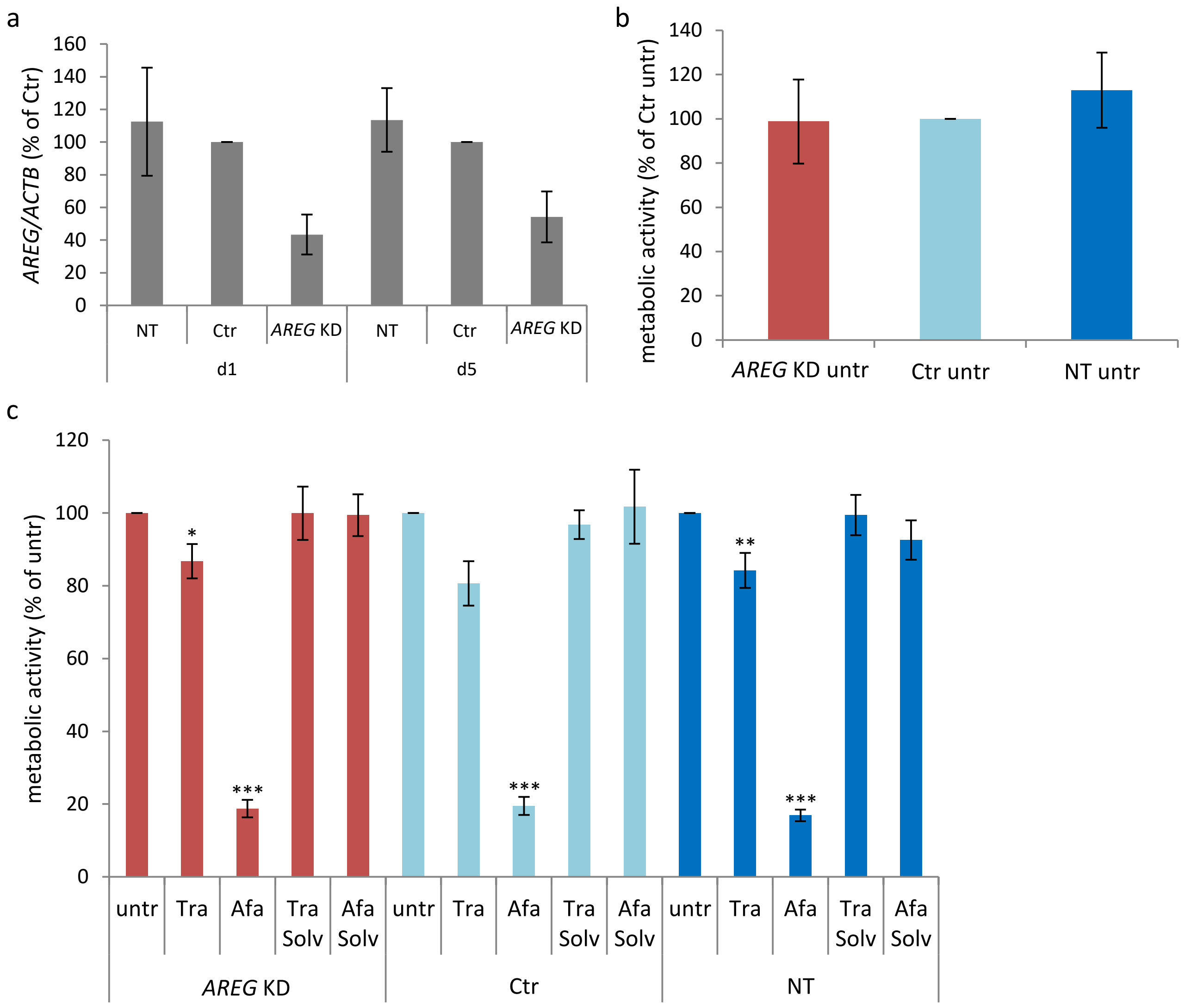

Figure S11: Effects of trastuzumab and afatinib on proliferation after *AREG* knockdown in NCI-N87 cells.
NCI-N87 cells were transfected with negative-control (Ctr) or *AREG* (*AREG* KD) siRNA. Non-transfected (NT cells) were used as control. The knockdown was checked on RNA level on day 1 (d1) and day 5 (d5) after transfection (a). The metabolic activity was measured by WST-1 proliferation assay for 72 h in the untreated (b) and treated (c) state. Cells were treated with 5 µg/ml trastuzumab (Tra 5), 20 µg/ml trastuzumab (Tra 20), 0.5 µM afatinib (Afa) or the corresponding solvents (Tra Solv, Afa Solv) for 72 h. Shown are the mean values from three experiments with standard deviation. Significant effects compared to untreated within a group (*AREG* KD, Ctr, NT) are indicated by ** $p<0.01$ or ***$p<0.001$ (one-sample t-test). Significant effects compared to Ctr or NT cells with the same treatment were not observed (two-sample t-test).

Figure S12

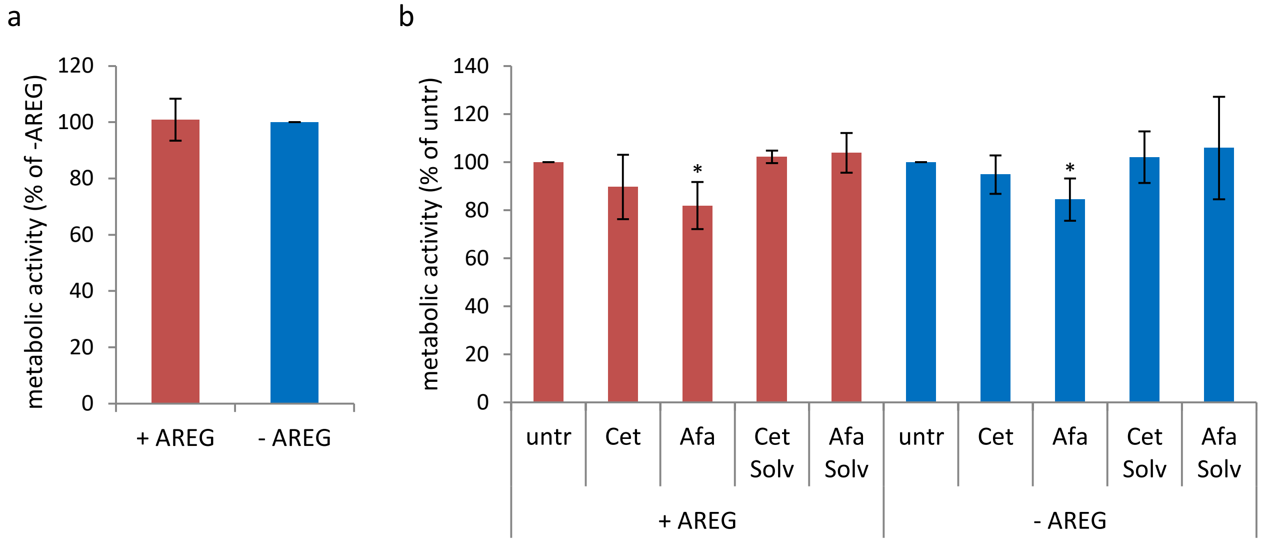

Figure S12: Effects of cetuximab and afatinib on proliferation after AREG stimulation in MKN1 cells.
MKN1 cells were stimulated with 15 ng/ml AREG (+AREG) or not stimulated (-AREG). The metabolic activity was measured by WST-1 proliferation assay for 72 h in the untreated (a) and treated (b) state. Cells were treated with 1 µg/ml cetuximab (Cet 1), 10 µg/ml cetuximab (Cet 10), 0.5 µM afatinib (Afa) or the appropriate solvents (Cet Solv, Afa Solv) for 72 h. Shown are the mean values from three experiments with standard deviation. Significant effects compared to untreated within a group (+AREG, -AREG) are indicated by *p<0.05 or (one-sample t-test). No significant effects compared to -AREG with the same treatment were observed (two-sample t-test).

Figure S13

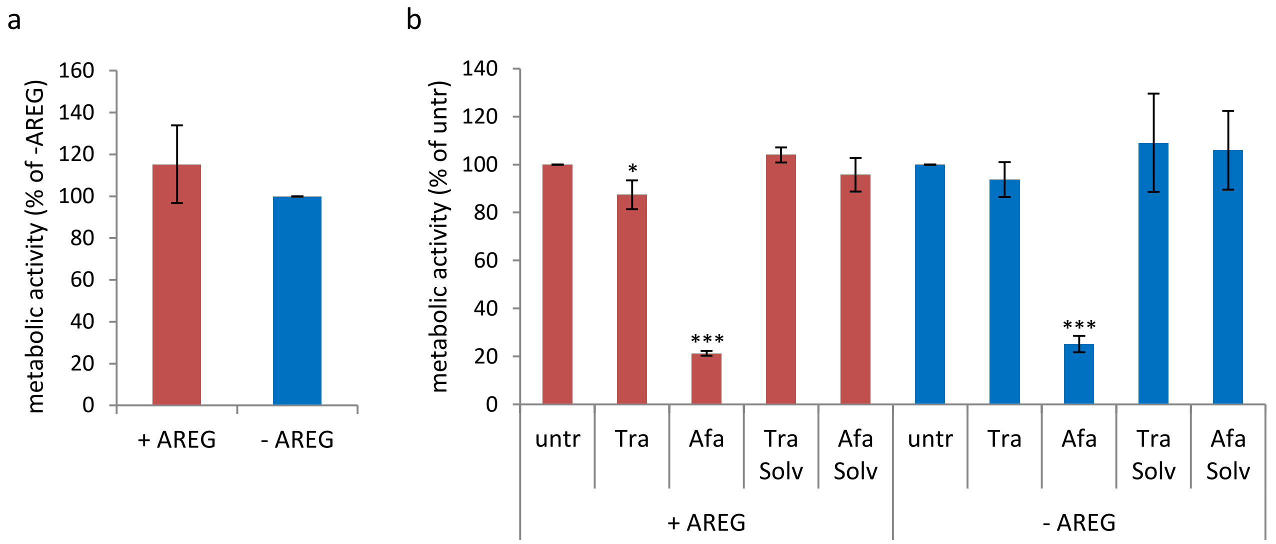

Figure S13: Effects of trastuzumab and afatinib on proliferation after AREG stimulation in NCI-N87 cells.
NCI-N87 cells were stimulated with 15 ng/ml AREG (+AREG) or not stimulated (-AREG). The metabolic activity was measured by WST-1 proliferation assay for 72 h in the untreated (a) and treated (b) state. Cells were treated with 5 µg/ml trastuzumab (Tra 5), 20 µg/ml Trastuzumab (Tra 20), 0.5 µM afatinib (Afa) or the appropriate solvents (Tra Solv, Afa Solv) for 72 h. Shown are the mean values from three experiments with standard deviation. Significant effects compared to untreated within a group (+AREG, -AREG) are indicated by *p<0.05 or ***p<0.001 (one-sample t-test). No significant effects compared to -AREG with the same treatment were observed (two-sample t-test).

Figure S14

a

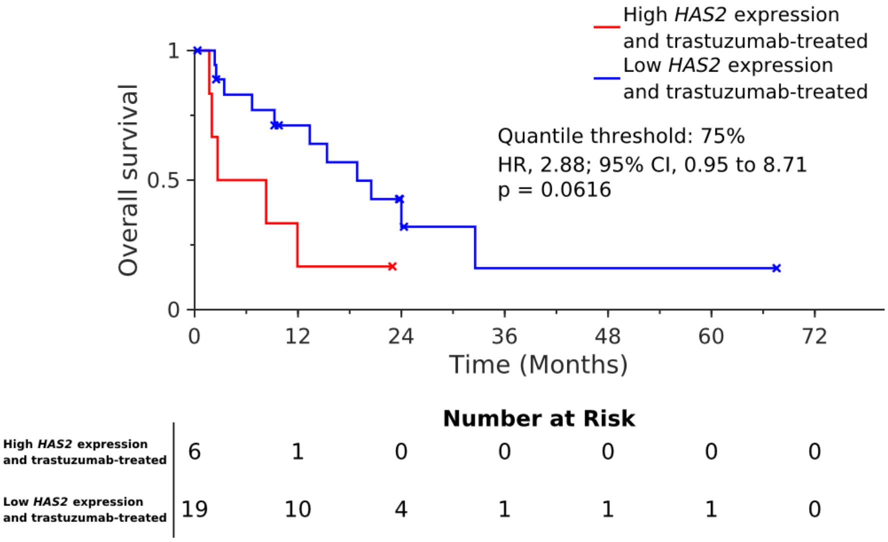

b

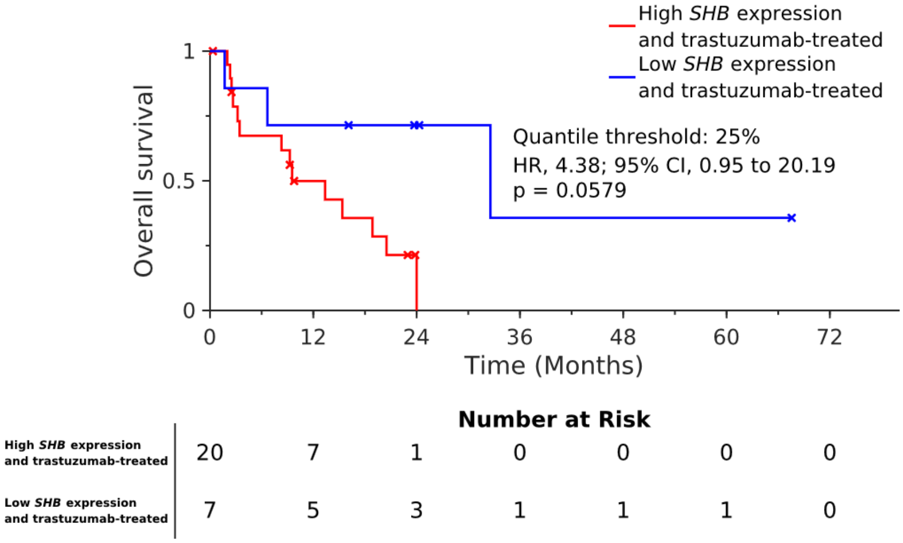

Figure S14: *HAS2* and *SHB* gene expression levels measured in tumor biopsies from trastuzumab-treated patients using quantile thresholds

a) Kaplan-Meier curves show a trend towards significance with respect to *HAS2* gene expression in patients receiving trastuzumab using quantile threshold 75% as cut-off. b) Kaplan-Meier curves show a trend towards significance with respect to *SHB* gene expression. in patients receiving trastuzumab using quantile threshold 25% as cut-off. The method employed to obtain the p values was the log-rank test.

Figure S15

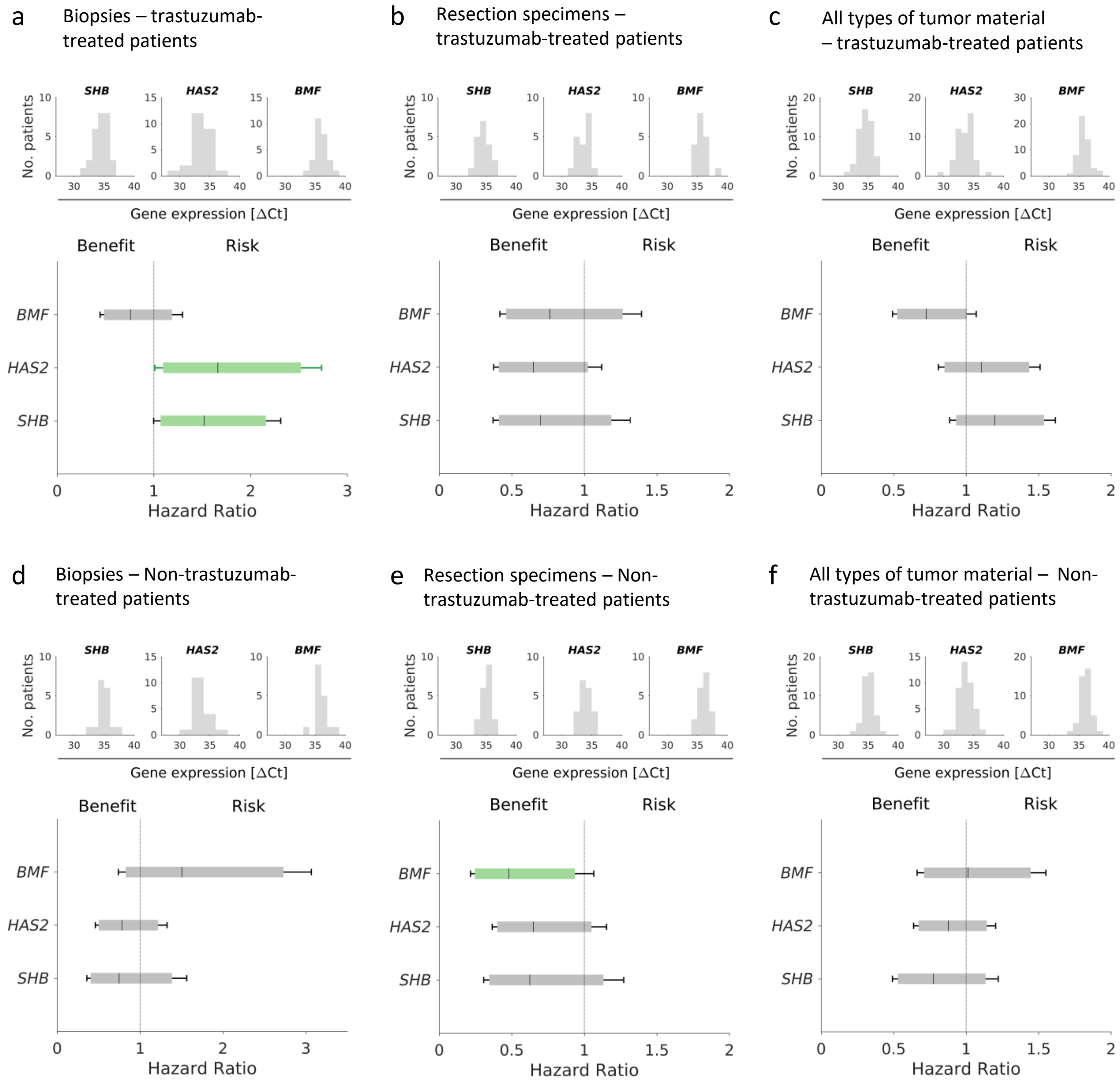

Figure S15: Distribution of RNA expression values and Hazard ratios obtained for *HAS2, SHB* and *BMF* gene expression.
a) Distribution of gene expression values and Hazard ratios (HR) obtained for *SHB* (HR 1.5; 95% CI, 1 to 2.31), *HAS2* (HR 1.7; 95% CI, 1.01 to 2.73) and *BMF* (HR 0.8; 95% CI, 0.44 to 1.3) in tumor biopsies from trastuzumab-treated patients. *SHB* and *HAS2* are risk factors for patient survival. b) Distribution of gene expression values and Hazard ratios (HR) obtained for *SHB* (HR 0.7; 95% CI, 0.37 to 1.31), *HAS2* (HR 0.6; 95% CI, 0.38 to 1.12) and *BMF* (HR 0.8; 95% CI, 0.42 to 1.39) in tumor resection specimens from trastuzumab-treated patients. c) Distribution of gene expression values and Hazard ratios (HR) obtained for *SHB* (HR 1.2; 95% CI, 0.89 to 1.62), *HAS2* (HR 1.1; 95% CI, 0.81 to 1.51) and *BMF* (HR 0.7; 95% CI, 0.49 to 1.07) in all types of tumor material – biopsies, metastases and resection specimens – from trastuzumab-treated patients. d) Distribution of gene expression values and Hazard ratios (HR) obtained for *SHB* (HR 0.7; 95% CI, 0.36 to 1.56), *HAS2* (HR 0.8; 95% CI, 0.46 to 1.33) and *BMF* (HR 1.5; 95% CI, 0.74 to 3.07) in tumor biopsies from non-trastuzumab-treated patients. e) Distribution of gene expression values and Hazard ratios (HR) obtained for *SHB* (HR 0.6; 95% CI, 0.31 to 1.27), *HAS2* (HR 0.6; 95% CI, 0.36 to 1.15) and *BMF* (HR 0.5; 95% CI, 0.22 to 1.07) in tumor resection specimens from from non-trastuzumab-treated patients. f) Distribution of gene expression values and Hazard ratios (HR) obtained for *SHB* (HR 0.8; 95% CI, 0.49 to 1.22), *HAS2* (HR 0.9; 95% CI, 0.64 to 1.2) and *BMF* (HR 1; 95% CI, 0.66 to 1.55) in all types of tumor material – biopsies, metastases and resection specimens– from non-trastuzumab-treated patients. The error bars show the 95% and boxes the 90% confidence interval. Green coloring shows significance.

Figure S16

a

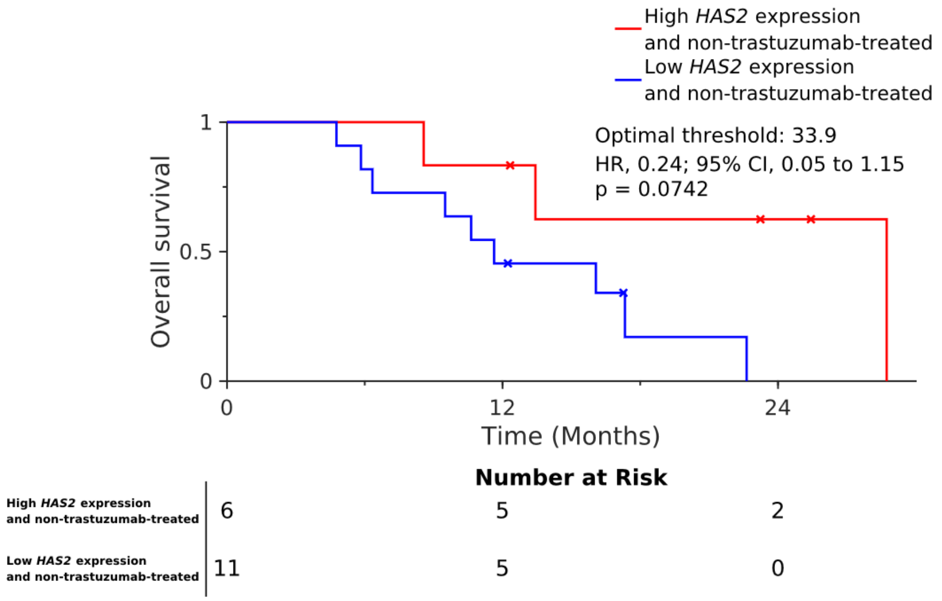

b

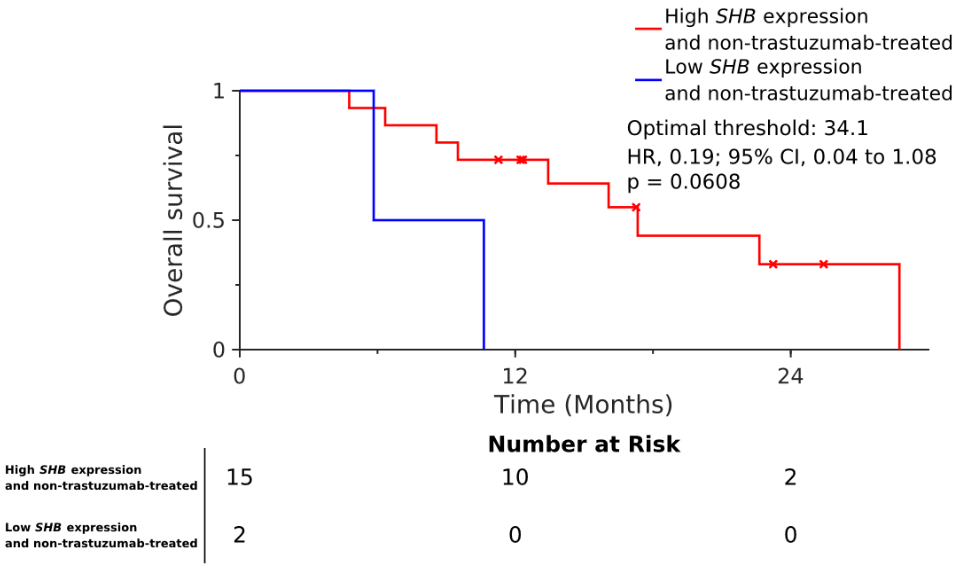

Figure S16: *HAS2* and *SHB* gene expression levels measured in tumor biopsies from non-trastuzumab-treated patients did not predict patient overall survival.

a) Kaplan-Meier curves show a trend towards significance with respect to *HAS2* gene expression in patients not receiving trastuzumab. b) Kaplan-Meier curves show a trend towards significance with respect to *SHB* gene expression. We refer to the main manuscript *Methods* for details in the threshold optimization for *HAS2* and *SHB* gene expression. The method employed to obtain the p values was the log-rank test.

Figure S17

a

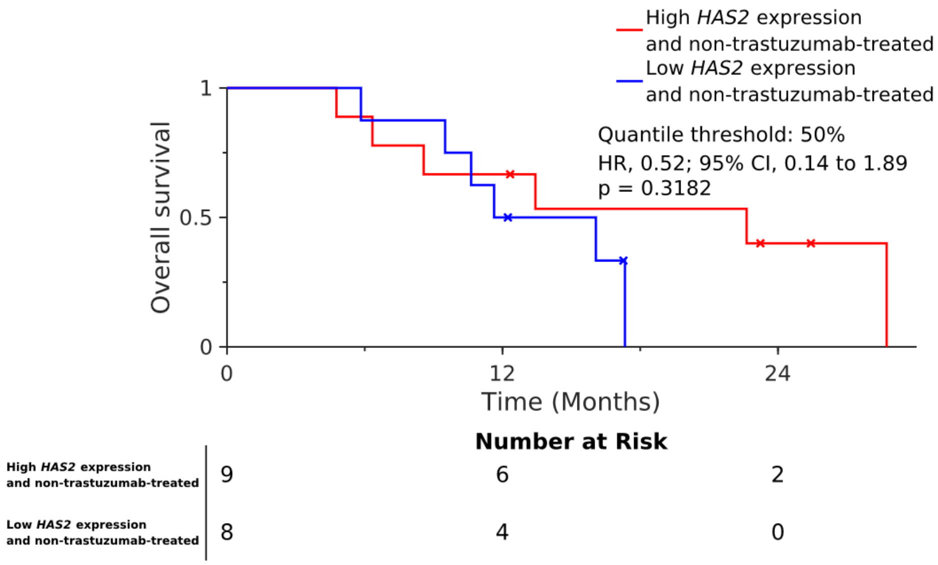

b

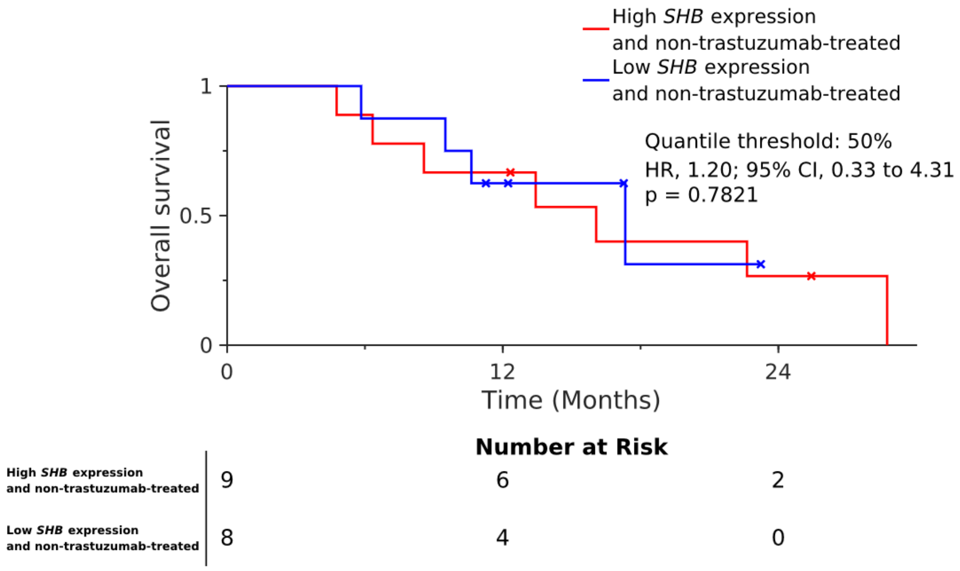

Figure S17: *HAS2* and *SHB* gene expression levels measured in tumor biopsies from non-trastuzumab-treated patients did not predict patient overall survival using quantile threshold 50%.

Kaplan-Meier curves show no significance with respect to *HAS2* (a) or *SHB* (b) gene expression in patients not receiving trastuzumab using quantile threshold 50% as cut-off. The method employed to obtain the p values was the log-rank test.
